# The Impact of Cultivation Management and Weed Control Systems of Very Early Potato on Weed Infestation, Biodiversity, and Health Safety of Tubers

**DOI:** 10.3390/life11080826

**Published:** 2021-08-12

**Authors:** Dominika Skiba, Barbara Sawicka, Piotr Pszczółkowski, Piotr Barbaś, Barbara Krochmal-Marczak

**Affiliations:** 1 Department of Plant Production Technology and Commodities Science, Faculty of Agrobioengineering, University of Life Sciences in Lublin, 20-950 Lublin, Poland; dominika.skiba@up.lublin.pl; 2 Experimental Station for Cultivar Assessment of Central Crop Research Centre, 21-211 Dębowa Kłoda, Poland; p.pszczolkowski.inspektor@coboru.gov.pl; 3 Jadwisin Research Center, Department of Potato Agronomy, Plant Breeding and Acclimatization Institute—National Research Institute, 05-140 Serock, Poland; p.barbas@ihar.edu.pl; 4 Department of Food Production and Safety, Carpathian State University in Krosno, 38-400 Krosno, Poland; barbara.marczak@kpu.krosno.pl

**Keywords:** biodiversity, biomarkers, food safety, herbicides, phytotoxicity, potato, variety, weed infestation

## Abstract

The aim of the research was to determine the impact of potato cultivation management and weeding systems on weed infestation and to evaluate the possibility of using biomarkers to assess consumer exposure to herbicide residues in potato tubers. The experiment was carried out in 2016–2018 in Central-Eastern Poland. The subject of research was the very early variety Lord. The experiment was established using the randomized block method in a split-plot design. The first order factor was cultivation management: (A) traditional and (B) under polyethylene sheeting (PE-sheeting) put “on flat”. The second-order factors were weed control systems: (a) mechanical (b) to (d)-chemical. The study determined the degree of damage to crops and weeds, fresh and dry weight of weeds, their number and floristic composition, and herbicide residues in tubers and in the soil. The fresh and dry mass of weeds was most effectively limited by mechanical and chemical treatment with the use of a preparation containing linuron. Managing potato cultivation with PE-sheeting and soil herbicides has proven to be safe for very early potato production. Used for pre-emergence care, the preparation containing linuron did not leave even trace amounts of this active substance in the tubers. The determined amount of the active substance fluorochloridon and clomazone was lower than the Maximum Residue Level (MRL) norm in the EU (European Union). As a result, the adopted, innovative management and weeding control systems in the cultivation of early potato varieties can be considered safe for the consumer.

## 1. Introduction

The potato (*Solanum tuberosum*, spp. *tuberosum* L.) is still one of the most important crops in the world, ensuring food and energy security for humans and livestock. Potato production has undergone many changes in the way this species is grown in different regions as various limiting factors need to be considered, including climatic conditions, water and nutrient availability, and light quality and quantity, to ensure favorable conditions for optimal crop growth and development. In particular, protected potato production enables growers to make changes to light quality or photobiology, which involves manipulating different wavelengths to modify the morphological characteristics of potato plants based on their specific light requirements [1]. Progress in the technology of growing potatoes for a very early harvest is best characterized by the correct selection of varieties; the use of certified, sprouted seed potatoes; the use of new methods and types of covers; the use of appropriate plant density and appropriate nutrition to the requirements; and a limited scope of plant protection, reducing the negative impact of pesticides on tuber quality. Potato cultivation under cover, especially in the changing climate of Central and Eastern Europe, allows, first of all, to extend the vegetation period of plants. Potatoes under covers can be planted much earlier and harvested longer. The covers also increase the humidity and air temperature in the crop, which gives the opportunity to cultivate varieties that are sensitive to changing weather conditions, especially those that are not resistant to ground frost in the spring. Potato is a species that tolerates frosts only down to −2 °C. Due to the fluctuations in air temperature in the spring in Central Europe, the cultivation of early potato varieties is safe under covers made of polyethylene foil or biofilm. It is best to locate them in an area with minimal weed infestation, because the possibilities of weed control under cover are very limited [1,2]. The use of plant protection products under cover, where it is not possible to use mechanical cultivation and protection techniques, raises the question of whether their residues do not pose a threat to potato consumers; hence, biomarkers were used in the studies to evaluate them.

Biomarkers as measurable changes in the cells of the body, and in them, biochemical processes are caused by specific ‘tags’ and at the same time are the evidence of toxins absorbed by the body. Based on exposure of biomarkers present in the body, one can obtain information regarding whether the body has been exposed to harmful factors [2]; whether the exposure, e.g., to xenobiotic caused biological effects and health risks (biomarkers effect) [3]; or whether the body is sensitive to the toxic effects of a given factor (biomarkers sensitivity) [4,5]. The substance chosen as a biomarker of exposure to a given xenobiotic should be easy to extract from the biological material and should show, in analysis, significant correlation with the size of the absorbed dose of xenobiotic. The biological monitoring is the study of the concentration of the toxic substance or metabolite produced by the action of biological material [6,7,8]. Knowing the path and speed of change that various substances in the body are subjected to, based on the analysis of biomarkers of exposure, one can assess the size of the absorbed dose of xenobiotic and the resulting health risks. Herbicides are an important xenobiotic as measures in the process of crop production. Thanks to them, it is possible to effectively combat weeds. However, long-term use of the same herbicides in the same areas may be the cause of weed resistance to the active ingredients of herbicides and the presence of residues of these substances in the soil, surface water, and groundwater in plant food products as well as in humans and animals [9,10,11,12,13,14]. What is important is the way of active substances of herbicides operate. Therefore, the following can be distinguished: inhibitors of acetyl-CoA carboxylase; acetolactate synthesis; photosynthesis; protoporphyrin oxidase, disrupting the synthesis of dyes, e.g., carotenoids; EPSP synthase inhibitors—5-enolpyruvate shikimic 3-phosphate synthase (EPSP synthase inhibitors); glutamine syntheses; dihydropteroate synthase inhibitor (DHP inhibitors), by interfering with microtubule formation; inhibitors of mitosis process; inhibitors of the formation of long-chain fatty acids; inhibitors of cell wall formation/cellulose synthesis, such as the destabilization of cell membranes; fat synthesis inhibitors; and inhibitors of auxin transport [3,4,14,15]. The HRAC organization (Herbicide Resistance Action Committee) has developed a classification of herbicides, depending on the mechanism of their action, and weed resistance to their effects [14,16]. The risk of the spread of herbicide-resistant weeds is largely dependent on the technology of cultivation of plants, the method of application of herbicides, and the degree of weed fields and weed biology [11,17,18].

Herbicides used in reducing weed infestation penetrate the tissues of plants and there interact with their life processes. They may inhibit or accelerate the growth of cells and influence the synthesis of proteins, cellulose, and the respiration of plants. They are not evenly distributed in plants; usually, they accumulate in their action place. Therefore, herbicide residues in plants are also not evenly distributed [8,19,20]. The residues in food products of active substances of herbicides used in plant production may be significant risk factors for lipid metabolism disorders, such as increased levels of triglycerides, total cholesterol, and low-density lipoprotein (LDL) fraction; lowering the level of lipoproteins in the blood; blood density (HDL), which leads to an increase in atherosclerotic changes in the aorta and coronary arteries; and diseases of the cardiovascular system. Therefore, they can cause greater DNA damage and chromosomal aberrations in human lymphocytes [3,10,15]. Herbicides used in plant cultivation get into surface water. This is a significant global issue [18,21]. Their use can lead to the occurrence of weed or insect resistance to their active substances. Herbicide resistance is the result of a combination of many internal and external factors related to the conditions of their application. Herbicides, especially selective herbicides, affect plants in different ways, most often by blocking the synthesis of amino acids, lipids, carotenoids, and other chemical compounds. On the other hand, other active substances block the transport of electrons in the photosynthetic system or cause disturbances in the plant growth cycle. Sulfonylurea compounds and imidazoles are ALS inhibitors, by which plants block the synthesis of leucine, isoleucine, and valine. An example of substances that affect the photosystem of plants are, for example, DCMU (3-(3,4-dichlorophenyl)-1,1-dimethylurea), which blocks the flow of electrons between the elements of the PS II and PS I photosystems due to the joined Q ubiquinone molecules. Changes in the structure of these molecules change their place of connection and render them unresponsive to DCMU. While the risks arising from the conditions of herbicide application can be changed by the user, the inherent risk is due to the interaction between certain characteristics of the pest, which is the purpose of the plant protection product [22]. The risk of practical resistance with unrestricted use and, if it is too high, how, and what modifiers can be introduced to reduce the risk to an acceptable level should then be determined [3]. The active substances contained in herbicides may easily penetrate chloroplasts, causing damage to photoreceptor II and the light energy collecting complex (LHC). According to Yefsah-Idres et al. [13], Agostinetto et al. [23], and Wu et al. [24], herbicides also interfere with the chlorophyll a: b ratio and reduce the activity of electron transporters. As a result, there are changes in the parameters of chlorophyll fluorescence and the induction of toxic enzymes. Hence, the purpose of the research was to demonstrate the possibility of biomarkers’ use to evaluate the exposure of potato damage to photoreceptor II and the light energy collecting complex (LHC) to herbicides. Currently, trends in sustainable agriculture aim at reducing the doses of herbicides used in order to reduce the degree of weed infestation to a level that is harmless to the crop and not to completely eliminate weeds, which is important not only for the regulation of weed infestation but also for the protection of the natural environment. The aim of the conducted research was to evaluate the influence of the active substance of herbicides on the possibility of reducing weed infestation in the cultivation of very early table potatoes grown under coverings assumed on “the flat” and the determination of their phytotoxic effects on plants.

The deadlines of herbicide application can be adapted to the periods of the greatest intensity of weed infestation in the potato cultivation [25]. The advantage of this approach to the use of herbicides is also the reduction of phytotoxic reaction of plants to herbicides, reduction of crop care costs, and significant reduction of biologically active substances residues in agricultural products while maintaining their high herbicidal effectiveness [26]. Hence, the aim of the research was to determine the phytotoxic effect of herbicides on weeds and crops, to assess the fresh and dry mass of weeds and their floristic composition, and to determine the herbicide residues in potato tubers and in the soil in the cultivation of a very early potato variety under PE-sheeting (polyethylene sheeting). Therefore, the main objective of the research was to demonstrate the possibility of using biomarkers to assess the exposure of potatoes to herbicides. 

Two research hypotheses were formulated. The first, alternative hypothesis assumes that the use of herbicides, on the one hand, will inhibit weed infestation in a canopy of a very early potato variety grown under a PE-sheeting. The second hypothesis assumes that the applied pre-emergence herbicides will contribute to the reduction of residues of biologically active substances in potato tubers while maintaining their high effectiveness, in view of the null hypothesis that the pre-emergence application of herbicides is not affected on the level of active substance residues in the tubers or of the reduction of weed infestation in the potato cultivation under a cover of polyethylene foil.

## 2. Materials and Methods

The studies were carried out in the years 2016–2018, in the station in Parczew, (51°38′ N; 22°54′ E; altitude: 148 m), on the Luvisols soil [27]. Parczew is located in the mesoregion of Central-Eastern of Poland, called the West Polesie. The field experiment was carried out by the method of random sub-blocks, in a dependent split-plot design, in the three replications. Surface of plots for harvest was 20 m^2^. The experimental factors were Factor I: Cultivation management: (a) with cultivation under a polyethylene sheeting (PE-sheeting), (b) traditional cultivation, without covers, and Factor II: weed control systems: (a) mechanical, (b) chemical using Afalon Dispersion 450 SC (active substance: linuron) at rate of 2 dm^−3^ × ha^−1^, (c) chemical using herbicide Racer 250 EC (active substance: fluorochloridon) at 2 dm^−3^ × ha^−1^, (d) chemical care using a mixture of herbicide Afalon Dispersion 450 SC + Command 480 EC (active substance: isoxazolidone clomazone) in an amount of 1 dm^−3^ + 0.2 dm^−3^ × ha^−1^. Treatments combined with spraying herbicides were applied only once during the vegetation period in the form of medium-drop spraying with 300 dm^−3^ × ha^−1^ of water. All plant protection products were used according to the manufacturer’s instructions provided on the label.

### 2.1. Characteristic of PE-Sheeting

The perforated polyethylene film used in the experiment had the following specifications: width: 3.6 m, color: transparent; type of perforation: 125 holes/m^2^, mesh diameter: 10 mm, thickness: 40 mic; UV stabilization, allowing for the full spectrum of sunlight. This cover provides plants with direct protection against temperature fluctuations during the day and at night, improves water management, and protects against moisture loss and spring frosts. PE-foil maintains UV resistance for up to 6 years, depending on the stabilizing additives used; it also has high light transmittance (up to 95%). The “on flat” PE-sheeting was applied after planting the tubers and applying weed protection on the formed ridges. The foil covering was performed with the use of a special aggregate, which spreads the cover and fixes it in the soil. The removal of the casing depended on the course of the weather, usually when all the plants had finished the emerging; if frost was still expected, the casing was left in the ridges to be reapplied. The edges of the foil were covered with earth and secured against tearing. Covering the field with foil allows increasing the soil temperature by up to 4 °C, compared to an uncovered field, which allows for bringing the planting date forward an average of 8–10 days.

### 2.2. Characteristic of Herbicides

**Figure 1:**
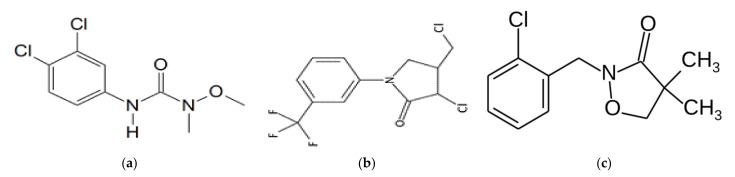
Chemical structure of selected active substances of herbicides: (**a**) linuron, (**b**) fluorochloridon, and (**c**) clomazone.

Afalon Dispersion 450 SC belongs to urea herbicides of the 3rd toxicity class. The chemical and physical characteristics of this preparation are shown in Figure 1a and Table 1. It is an inhibitor of photosynthesis and electron transport [28].

Racer 250 EC is a toxic class IV herbicide. The chemical and physical characteristics of this herbicide are shown in Figure 1b and Table 1 (Figure 1b, Table 1). Fluorochloridon is a member of the pyrrolidines, (trifluoromethyl) benzenes, and the organochlorine compound. It acts as an inhibitor of carotenoid biosynthesis, an agrochemical, and an herbicide [29]. Command 480 EC is a product of the 3rd toxicity class. The physico-chemical characteristics of this preparation are presented in Figure 1c and Table 1. This herbicide mainly blocks the synthesis of carotenoids [30].

### 2.3. Characteristic of Variety

Lord-a variety of great economic importance popular in Poland. The characteristics of this variety are included in Table 2.

### 2.4. Field Research

The forecrop of potato was barley. Fertilization and protection of potato against weeds and pests were the same for all objects of experience. The manure was applied at a dose of 25 t × ha^−1^ in the autumn, and in the spring mineral fertilizers were applied in quantities of 80 kg × N, 42 kg × P, and 114 kg × K × ha^−1^. Sprouted tubers of a very early cultivar of Lord were planted in the period 15–18 April in the spacing of 67.5 cm × 37 cm. After planting, the potato tubers were carefully ridging and sprayed, and an appropriate herbicide was applied to the objects with chemical weed control systems. After this treatment, the ridges were covered with covers and their edges covered with earth. The covers were removed after the end of ground frosts, but not earlier than at the plant height of 15–20 cm (from 25 May to 10 June, depending on weather conditions). In the objects with mechanical weed control, harrowing with a light ridging and covering it twice was carried out. Plant protection treatments against the *Leptinotarsa decemlineata*, *Phytophthora infestans,* and *Alternaria solani* were carried out in all the objects following the principles of Good Agricultural Practice [32] (Table 3). The tubers were harvested during Phase 99 according to the Biologische Bundesanstalt, Bundessortenamt und Chemische Industrie (BBCH) scale [33].

### 2.5. Sampling and Soil Assessment Methodology

Before setting up the experiment and in each year of conducting the experiment, soil samples were collected from a 0–20 cm layer in 5 replications. In each replication, the soil samples were taken from 5 different places from the arable layer (0–20 cm depth) using a Nekrasov auger. The representative soil samples were taken to determine the content of humus, soil pH, and the content of basic macro and micro-elements in the soil. The soil samples were analyzed for soil particle size using the laser method [34], pH in 1 mol KCl dm^−3^ [35], and organic carbon content (Corg) by the Tiurin method [36], and on its basis, the content of humus in the soil was determined [37]. The content of available phosphorus [38], potassium [39], and magnesium [40] was also determined in the soil. The chemical and physicochemical traits properties of the soil were determined by the following methods: soil granulometric composition was determined by laser diffraction [41]; pH in the suspension of 1 mol KCl dm^−3^; pH in H_2_O in a suspension by the potentiometric method [35]; organic carbon content (Corg.) by the Tiurin method [36]; available magnesium quantity by the Schachtschabel method [40]; and the content of absorbable forms of potassium and phosphorus by the Egner-Riehm method [38,39]. The results of soil analysis were valued according to limit numbers developed by the Institute of Soil Science and Plant Cultivation, National Research Institute in Puławy (Poland) [42].

### 2.6. Weed Infestation

During plant growth and development, observation of potato development and plant damage caused by herbicides according to the 9° EWRC scale (European Weed Research Council) was carried out every 7 days. It concerns a different assessment for weeds and another for phytotoxic effect on a crop plant: for a crop plant, 1 means no damage and 9 means complete destruction of the cultivated plant; for weeds, 1 means complete destruction of weeds and 9 means no action on weeds. The weed infestation was determined using two methods: aerophyte-sociological and frame methods. The aerophyte-sociological method was used to determine the percentage of soil coverage with cultivated plants and monocotyledonous and dicotyledonous weeds. The assessment of weed infestation was carried out on an area of 0.5 m^2^, and using a frame measuring 33.4 cm × 150 cm the following were determined: the fresh and air-dry weight of weeds, the number of the mono- and dicotyledonous weeds, and the floristic composition of weeds. Assessment of weed infestation was performed on three dates: before and after row closure and before harvesting of the potato [43].

### 2.7. Tuber and Soil Sampling

Tuber and soil samples were collected in accordance with the applicable standards [44,45,46] during potato harvesting. The pre-prepared samples (cleaned, shredded, and mixed) were stored until chemical analyses were performed in closed plastic containers at a temperature of minus 18 °C. The obtained test results were compared with the maximum permissible residue levels (MRLs) in force in the European Union [47].

### 2.8. Research on Herbicide Residues

The analytical process for determining residues consisted of three stages: extraction of analyses from the sample, purification, and quantification of the extract. The determination of herbicide residues was carried out using high-performance liquid chromatography (HPLC) with UV detection. The analytical procedures used for determining residues were based on standards [45,46,47]. Plant analyses were performed using the accredited chromatographic and spectrophotometric method, according to the PN-EN ISO/IEC 17025 [48] standard, enabling simultaneous detection of many compounds. The analysis of samples was performed at the certified analytical laboratory of the Faculty of Chemistry of Maria Curie-Skłodowska University in Lublin (Poland). The samples of potato tubers, 500 g, were washed in running water, blotted, cut into quarters, and then ground in a laboratory mill. The primary sample was divided into secondary samples, weighing 100 g, which were then homogenized for 10 min with the addition of 100 mL of acetone p.a. After decanting, the precipitate solution was filtered through a pad of 20 g of anhydrous sodium sulfate p.a. remaining after decantation; the precipitate was poured over 50 mL of acetone and again homogenized for 10 min and filtered through sodium sulfate [49]. The extracts were combined, and then 300 mL of distilled water and 30 mL of saturated sodium chloride p.a. were added to 150 mL of the extract and then extracted in a liquid–liquid of two portions (70 mL) of dichloromethane, using a separator funnel containing 500 mL, and the lower phase was collected. The combined final extract was dried with anhydrous sodium sulfate. Then, the decanted extract was evaporated to dryness in a water bath under a vacuum. The dry residue in 2 mL of dichloromethane was dissolved. The collected concentration was 50 g of the starting sample per 1 mL of the final solution. Thus prepared, the extract was further purified on an SPE column cartridge conditioned with 5 mL of a solution of acetone (10% *v*) in dichloromethane (90% *v*). It was applied to the column 1 mL of purified extract, sipping it slowly. The adsorbed on stanchion pesticides eluted with 4 mL of acetone in dichloromethane. The final eluate was analyzed using gas chromatography using a capillary column and an ion trap by a mass spectrometer as the detector. The metered sample eluate was 1 μL [49]. All methods were validated, mainly with the use of gas chromatography techniques with MS detection (Varian CP-3800, Varian Saturn 2200 GC/MS/MS, Agilent, Santa Clara, CA, USA). The test results were confirmed in accordance with the European Commission guidelines [50]. The obtained results were compared with the maximum permissible levels of pesticide residues (MRLs) in force in Poland [51,52,53]. All of the necessary statistical analyses were performed in Statgraphics Centurion version XV.

### 2.9. Soil Conditions

Field experiments were carried out at Parczew (51°38′ N; 22°54′ E; altitude: 148 m) on Luvisol soil [27]. The experiment was carried out on the sandy loam soil type. According to the percentage content of sand, silt, and loam fraction, this is a granulometric subgroup—clay sand (light soil), on sandy loam soil, medium or strong, good rye complex. The fraction of sand was 66.96%, the dust fraction was 30.58%, and the loam was 2.45% (Table 4). Due to the percentage of sand, silt, and clay fractions, it is a granulometric subgroup—light silty soil (medium soils), soils made of light clay sands—these soils belong to the slightly acidic good rye complex [37]. 

The humus content in the arable layer of the soil was low and ranged from 0.94–1.06% (Table 5). The content of assimilable macronutrients in the soil was as follows: w phosphorus and magnesium—medium (21.0 mg P_2_O_5_ × 100 g^−1^ soil, 7.0 mg × Mg 100 g^−1^ soil), w available potassium—low (11.9 mg K_2_O × 100 g^−1^ soil). The content of assimilable microelements in the soil substrate was also diversified over the years of the research and was as follows: the abundance of copper, manganese, and iron in the soil was average (7.02 mg Cu, 274 mg Mn, and 3762 mg Fe × kg^−1^ of soil). The abundance of available boron in the soil was high (6.17 mg B × kg^−1^ soil). The average soil acidity in the KCl solution in 2016 and 2017 ranged from 5.92 to 5.77 pH; These values made it possible to classify the soil under test as slightly acidic, while in 2018, the soil under the experiment was neutral (pH 6.6) (Table 4).

Ranges of values of this index were classified as follows: month extremely dry—≤k 0.4; month very dry—0.7 ≤ k < 0.4; month dry—1.0 ≤ k < 0.7; month rather dry—1.3 ≤ k <1.0; month optimal—1.6 ≤ k < 1.3; month rather humid—2.0 ≤ k < 1.6; month wet—2.5 ≤ k < 2.0; month very humid—3.0 ≤ k < 2.5; and month extremely humid—3.0 > k.

### 2.10. Meteorological Conditions

Meteorological conditions in the years of research varied. The year 2016 can be classified as humid. The year 2017 was one of the least favorable, with excess rainfall in May and June but significant rainfall shortages in July and August, decisive for yield, and 2018 was average both in terms of temperature and precipitation (Table 5). 

### 2.11. Statistical Analysis

Statistical analyses were based on three-factor analysis of variance (ANOVA—analysis of variance). The significance of the sources of variation was tested with the Fischer–Snedecor “F” test, and the value of honest significant difference (HSD) was assessed by multiple tests of T-Tukey, with the assumed significance level α = 0.05. Normalizing transformations were used for features expressed as a percentage close to 0 or 100. The models of analysis of the variance with the main effects of the factors studied and their interactions were used. The detailed analysis only dealt with the main effects. The calculations were made with the SAS Enterprise 4.2 program [54]. 

T-Tukey’s multiple comparison tests enabled detailed comparative analyzes of averages by isolating statistically homogeneous medium groups and determining the so-called smallest significant mean differences (LSD), which in Tukey’s tests are marked by HSD [54]. In the case of detailed analyzes based on T-Tukey’s multiple tests, the significance level α = 0.05 was assumed. Letter indicators at averages determine the so-called statistically homogeneous groups. The occurrence of the same letter pointer at averages (at least one) means that there is no statistically significant difference between them. The sizes of HSD perform an auxiliary role, allowing the quantification of the differences between means in a quantitative way.

## 3. Results

### 3.1. Weed Infestation

#### 3.1.1. Soil Coverage by Arable Crops and Weeds

The soil coverage with potato plants was 92.1% on average, 5.9% with monocotyledonous weeds, and 1.9% with dicotyledonous weeds. The use of PE-folie as covers in potato cultivation contributed to an increase in soil coverage by both monocotyledonous and dicotyledonous weeds.

**Figure 2:**
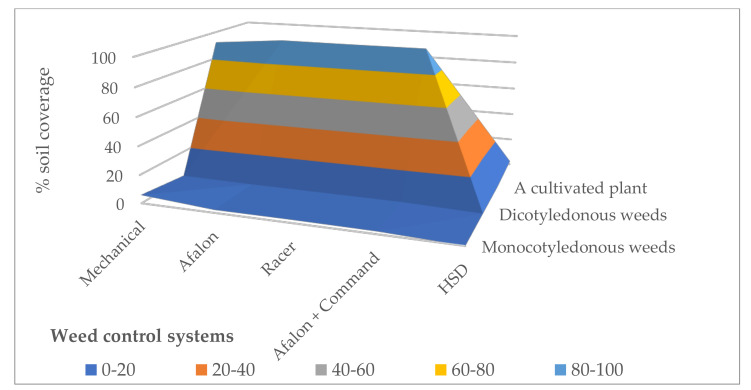
Covering the soil with monocotyledonous and dicotyledonous weeds and cultivated plants, depending on the potato weed management control (2016–2018).

The coverage of soil with monocotyledonous weeds was significantly differentiated from the weed control systems. Monocotyledonous weeds’ best was limited to the chemical weed control systems compared to the mechanical system. Dicotyledonous weeds similarly restricted all weed control systems. Coverage of soil with cultivated plants was also significantly differentiated by weed control systems. The lowest soil coverage with arable crops was found in the facility with a mechanical method of weed infestation regulation. The methods of chemical regulation of weed infestation in potato cultivation turned out to be homogeneous with respect to each other in terms of the size of this feature, but all of them turned out to be significantly better than mechanical potato care (Figure 2).

**Figure 3:**
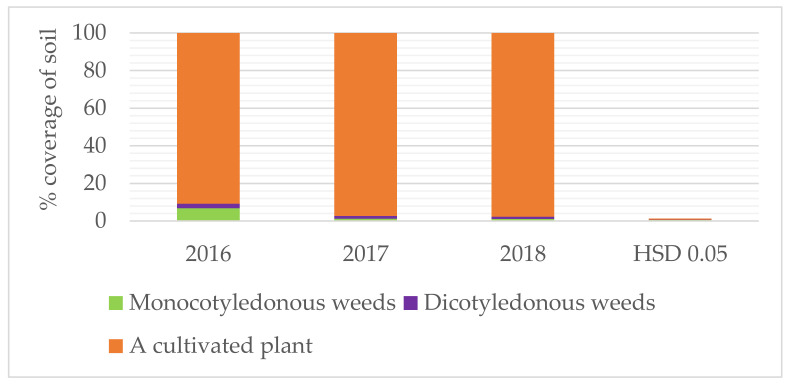
Covering the soil with weeds and crops, depending on the years.

Meteorological conditions in the research years significantly modified soil cover, both by crops and weeds. The highest soil coverage by both weed groups, and the smallest by arable crops, was recorded in 2016, with the lowest amount of rainfall during the potato growing season, which indicates greater resistance of weeds to stressful conditions. The lowest soil coverage with both monocotyledonous and dicotyledonous weeds, and the largest with crops, was observed in 2018, with a distribution of rainfall favorable for potatoes; these values turned out to be homogenic in 2017 (Figure 3).

#### 3.1.2. Reaction of Plants to Herbicides

The mean degree of herbicide damage to cultivate plants was 1.3° according to EWRC 9° (Table 6). The greatest damage to crops was observed after the application of the fluorochloridon. They showed chlorotic spots on the leaves and discoloration of their nerves. Much smaller phytotoxic damage to potato plants was observed after the application of the following: linuron and mixtures: linuron + clomazone. The greatest phytotoxic damage to potato plants was observed in 2016 with a late but cool and wet spring, and in a very wet and cool 2017. Stronger phytotoxic symptoms on potato plants in these years should be explained by a large amount of rainfall (month of May) after chemical treatments, which resulted in greater uptake of the active substance of the preparations than in 2018, with an average humid May. As the crop grew, the phytotoxic symptoms gradually disappeared.

The mean degree of damage to dicotyledonous weeds was 7.5° in a 9° European Weed Research Council (EWRC) scale (Table 6). Cultivation management using polyethylene film covers did not increase the degree of damage to this group of weeds compared to conventional cultivation. However, the potato care methods had the greatest impact on the value of this feature. The greatest damage to dicotyledonous weeds was observed after the application of the herbicide Afalon Dispersion 450 SC + Command 480 EC, less after the application of Racer 250 EC, and the lowest after the application of the herbicide Afalon Dispersion 450 SC. Racer 250 EC caused the greatest damage to this group of weeds, followed by Afalon Dispersion 450 SC + Command 480 EC. The highest degree of weed damage in this group was observed in 2016 with a very wet May, and the lowest in 2018 with an optimal amount and distribution of rainfall in the vegetation period. The strongest phytotoxic symptoms were observed on dicotyledonous weed plants in the first observation period. As the plants developed, these symptoms gradually disappeared.

The mean degree of damage to monocotyledons was 7.3° on the 9° EWRC scale (Table 6). The use of covers in potato cultivation contributed to the reduction of the degree of damage to monocotyledonous weeds compared to the traditional technology. All methods of chemical care increased damage compared to mechanical care. The greatest phytotoxic damage was observed on monocotyledonous weeds after the application of the herbicide Afalon Dispersion 450 SC + Command 480 EC, and then after the application of preparation Racer 250 EC, although the difference between these treatments turned out to be insignificant, and the least damage was caused by the application of Afalon Dispersion 450 SC. The highest symptoms of phytotoxic damage in this group of weeds were observed in 2016 in a very humid May, and the lowest in 2018 in a warm, but not too humid, period of plant emergence. The greatest reaction of monocotyledonous weeds to herbicides was observed in the first observation period, and then, with the development of plants, these symptoms systematically disappeared.

#### 3.1.3. Fresh and Air Dry Mass of Weeds

The average weight of the weeds was 96 g × m^−2^. The lowest weed mass was recorded before the rows were closed, and the highest mass before the potato maturation, which was caused by secondary weed infestation (Table 7). 

The cultivation management of potato did not significantly affect the size of the fresh and dry mass of weeds, while the weed control system modified them. Chemical weed control with herbicide Afalon Dispersion 450 SC resulted in a significant reduction in the fresh weight of weeds compared to mechanical care. The Afalon Dispersion 450 SC + Command 480 EC herbicide blend in the control of weed infestation had a similar effect on weed weight reduction as mechanical control. The lowest weight of weeds was recorded in 2017, the coolest and at the same time the most humid, and the highest in 2016, with late and cold spring and the lowest rainfall. In 2016, the weight of weeds was reduced the most using the mechanical and chemical method of tending Afalon Dispersion SC, and in 2018, with the most favorable rainfall distribution, with the herbicide Racer 250 EC (Table 7). 

The air-dry weight of weeds was on average 36 g × m^−2^ and the highest before the potato were harvested (Table 7). The management of potato cultivation did not significantly differentiate the dry matter of weeds. A significant increase in this value was observed only before the potatoes ripened. The applying of Racer 25 EC resulted in a significant increase in the dry weight of weeds compared to the control object and with other weed control systems. However, the decreasing effect of care with Racer 25 EC was noted only in the period before tuber harvest (secondary weed infestation). The lowest dry mass of weeds was recorded in 2017, with the air temperature below the norm and the total rainfall significantly exceeding the long-term average. The highest value of this feature was observed in 2018, with warm spring and summer and evenly distributed rainfall (Table 7).

#### 3.1.4. Number of Weeds

The average total number of weeds was 46 m^−2^. Cultivation management did not significantly affect the number of weeds, both monocotyledonous and dicotyledonous (Table 8).

Weed infestation control systems turned out to be a factor that significantly differentiated weed infestation. The number of monocotyledonous weeds was most limited by the treatment with Afalon 450 S.C., while the number of dicotyledonous weeds was the most decreased by the Afalon 450 S.C. + Command 480 EC compared to other weeds control systems. In the case of monocotyledonous weeds, the response of the weeds to control systems depended on the time of observation. The use of Racer 250 EC to regulate weed infestation was the least effective in the second and third observation periods (Table 8).

Meteorological conditions in the research years also interacted with the number of mono- and dicotyledonous weeds. The smallest weed infestation was observed in 2017, and the largest in 2016, in cold spring. In 2018, despite the high number of weeds marked before the potato rows were closed, the weed infestation dropped drastically in the second and third terms, which resulted from droughts at the later vegetation date (Table 8).

#### 3.1.5. Floristic Composition of Weeds

In the conducted research, seven species of monocotyledonous weeds and twenty species of dicotyledonous weeds were recorded (Table 9).

The most abundant monocotyledonous weeds were *Echinochloa crus-galli*, *Setaria glauca*, and *Setaria viridis*, and sporadically *Avena fatua* and *Poa annua*. The highest number of Echinochloa crus-galli was found before the row closure of potato plants. *Setaria viridis* and *Setaria glauca* were most numerous before the harvesting of potatoes, which results from the developmental biology of these species (Table 9).

The cultivated technology of potato significantly modified the number of mono- and dicotyledonous species. The cultivation under cover with PE-sheeting increased the quantity only of two species of dicotyledonous weeds (*Anthemis arvensis* and *Cirsium arvense*) while the traditional technology contributed to the greater of two monocotyledons species (*Echinolchloa cruss-galli, Setaria glauca*) and one of the species from the dicotyledonous class (*Viola arvensis*) (Table 9).

*Echinochloa crus-galli* species was most effectively fought by chemical with herbicide Afalon Dispersion 450 SC, compared to mechanical treatment. The applying of the Afalon Dispersion 450 SC + Command 480 EC herbicide mixture significantly fought *Echinochloa crus-galli*, while the use of the Racer 250 EC herbicide did not reduce weed infestation for this species (Table 9).

Meteorological conditions in the research years turned out to be the factor that differentiated the number and floristic composition of weeds to the greatest extent. In wet and quite cold 2017, only three species of monocotyledonous weeds appeared, while in warm 2016, as many as seven species appeared, including *Echinochloa crus-galli* in great numbers. Only *Echinochloa crus-galli, Setaria glauca,* and *Setaria viridis* remained in the secondary weed infestation. Of the class of dicots, the most common weed species were *Anthemis arvensis* and *Chenopodium album. Viola arvensis, Veronica hederaefolia, Cirsium arvense,* and *Stellaria media* appeared much less frequently. The remaining species of the dicotyledonous class occurred sporadically (Table 9).

Mechanical weed control only significantly fought the occurrence of *Anthemis arvensis*. The use of the herbicide Afalon Dispersion 450 SC successfully reduced the number of weed species such as *Stellaria media* and *Centaurea cyanus*. However, it turned out to be ineffective against *Anthemis arvensis, Polygonum convolvulus*, or *Veronica hederifolia*. The use of preparation Racer 250 EC in potato care completely eliminated *Polygonum lapathifolium* and effectively limited *Polygonum convolvulus* and *Viola arvensis*. The use of a mixture of Afalon Dispersion 450 SC + Command 480 EC preparations significantly reduced the occurrence of *Chenopodium album, Cirsium arvense, Spergula arvensis,* and *Vicia tetrasperma* (Table 9).

Meteorological conditions in the research years also modified the floristic and quantitative composition of dicotyledonous weeds. In 2016, warm and sunny but with little rainfall, a greater number of weed species were found, such as *Anthemis arvensis, Stellaria media, Veronica hederifolia, Vicia tetrasperma*, and *Viola arvensis*. In wet and cold 2017, the greatest numbers were *Cirsium arvense, Polygonum lapathifolium, Polygonum aviculare, Spergula arvensis, Raphanus raphanistrum*, *Rumex acetosella*, and *Vicia hirsuta*. Before the potato rows were closed, the most numerous were *Chenopodium album, Raphanus raphanistrum,* and *Polygonum convolvulus*. Immediately after the rows were closed, the most difficult species turned out to be *Cirsium arvense*, and before the potato harvest, *Anthemis arvensis* (Table 9).

### 3.2. Herbicide Residues in the Soil

Minimum residues of linuron, fluorochloridon, and clomazone were detected in the soil in which potatoes were grown. The level of residues depended on the type of active substance and meteorological conditions during the growing season. In the soil, the lowest levels of the active substance of the clomazone and the highest levels of linuron were detected. The residual of linuron and fluorochloridon were homogeneous in terms of the value of this feature (Table 10). The meteorological conditions in the years of the study had the greatest impact on herbicide residues in the soil. The least residue of herbicides was found in humid 2017, the highest in rather dry 2016 (Table 10).

### 3.3. Herbicide Residues in Potato Tubers

The active substances were determined in samples of plant material and in soil samples. The obtained results were compared with the maximum permissible residue levels (MRLs) in force in Poland [51,52,53]. The value of residues of active herbicides in potato tubers was similar: linuron and fluorochloridon—0.01 mg kg^−1^ and clomazone—0.02 mg × kg^−1^ MRL. The level of herbicide residues was assessed on the basis of the Commission Regulations (EU) [52,53,54].

The chromatogram of the individual herbicides wavelength is given in the Figure 4, Figure 5, Figure 6, Figure 7, Figure 8, Figure 9, Figure 10, Figure 11, Figure 12, Figure 13, Figure 14 and Figure 15.

**Figure 5:**
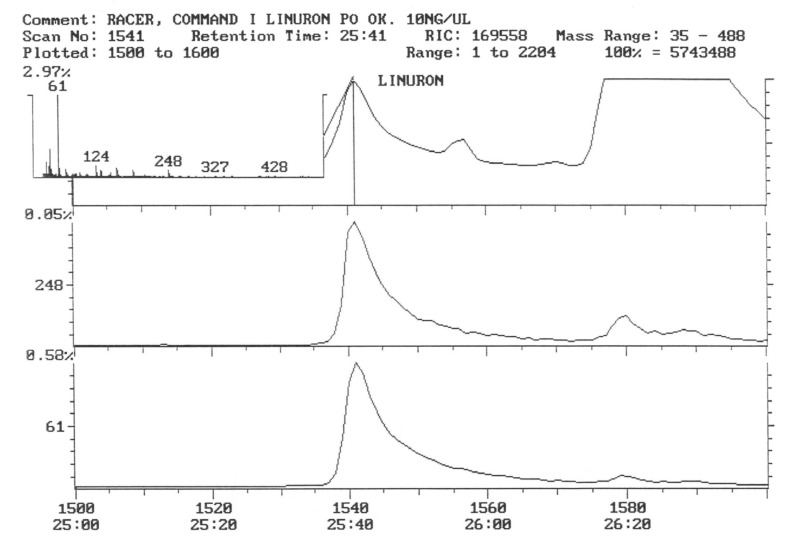
Chromatogram of the wavelengths of the individual active substances.

During the analysis of samples, the active substance, linuron, was not detected. There has been a peak, a characteristic ion of 61, but derived from ion peak 60, which is an unknown substance ballast with the same shape and size (Figure 5). It must, therefore, be assumed that even if there are traces of linuron, at this level of concentration, they are undetectable by this method.

**Figure 6:**
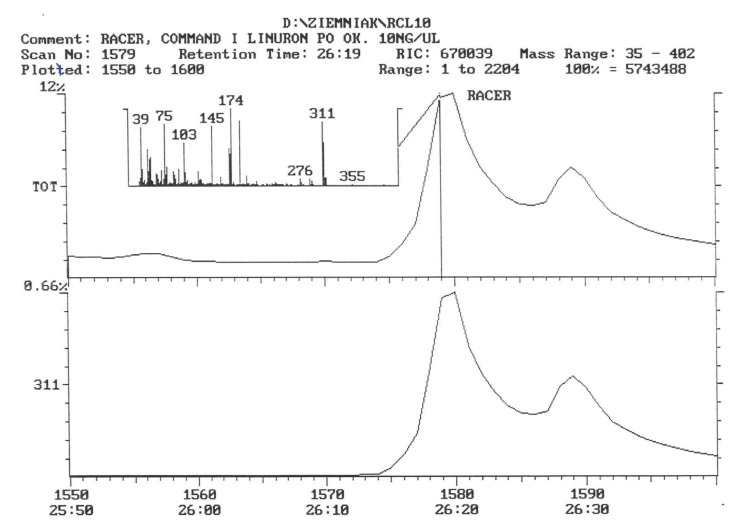
Chromatogram of a sample treated with Racer 25 EC, 2016.

The active substance, which is fluorochloridon, was not found in tubers from the site where the Racer 25 EC herbicide was applied to the soil (Figure 6).

**Figure 7:**
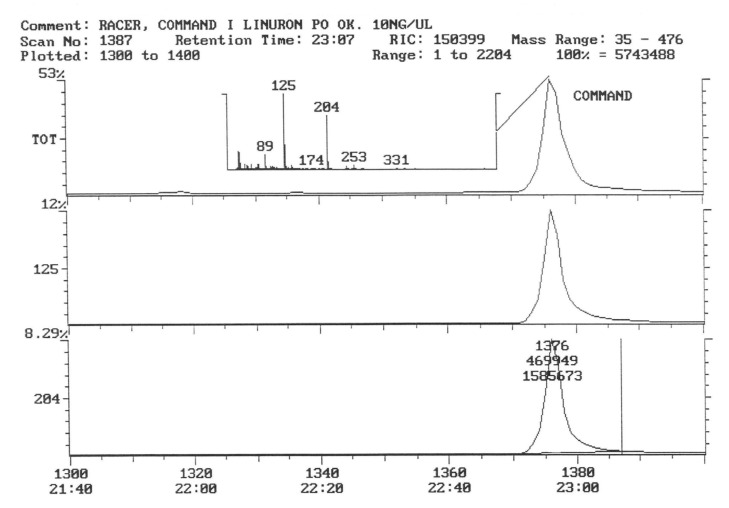
Chromatogram of the sample treated with Afalon 450 SC + Command 480 EC preparations.

In the samples from the combination sprayed with the mixture of preparations, Afalon 450 SC + Command 480 EC, containing linuron and clomazone as active substances, a substance with the characteristic ion 204 was detected (Figure 7). This indicates the presence of clomazone. The concentration of clomazone was calculated on the basis of the standard chromatogram, and it was 0.0002 mg × kg^−1^ of the wet potato tuber sample. This content is much lower than the MRL (Regulation EC No 396/2005396/2005 of the European Parliament and of the Council of 23 February 2005 on maximum residue levels of pesticides in or on food and feed of plant and animal origin and amending council directive 91/414/EEC).

**Figure 8:**
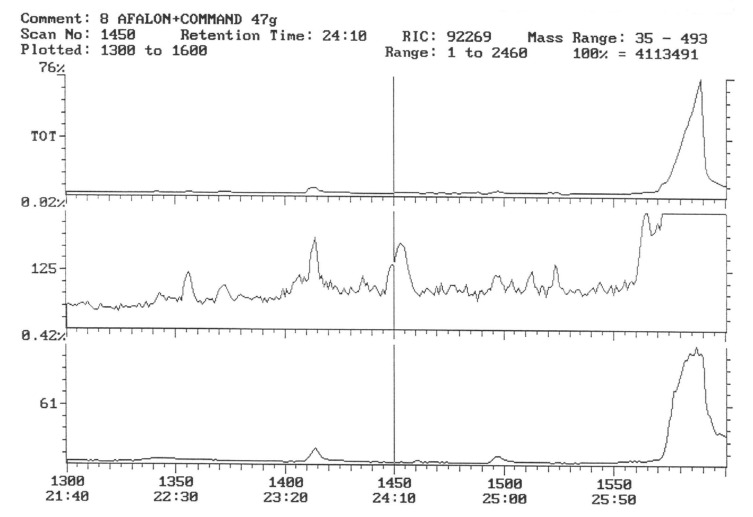
The chromatogram of the samples treated with active substance linuron+ clomazone.

In 2017, no pesticide residues were found in tubers. This was probably due to the fact that during the potato vegetation period in 2017, there was very high rainfall, much higher than in many years, which could have contributed to the washing away of any residues of plant protection products (Figure 8).

**Figure 9:**
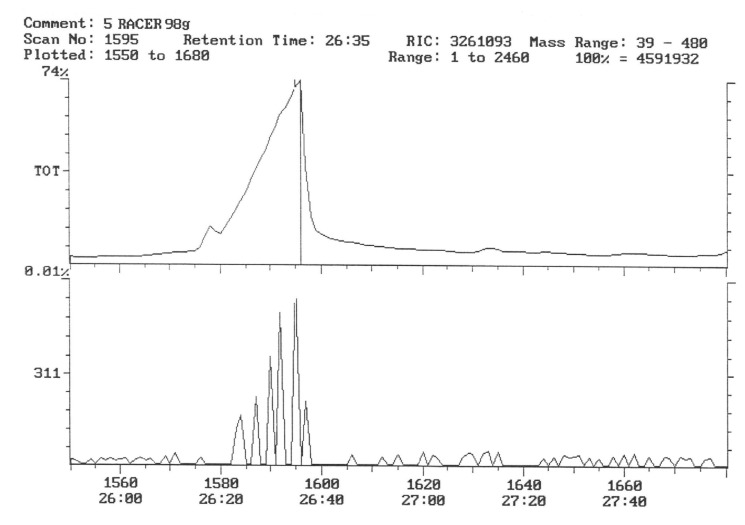
The chromatogram of the sample treated with herbicide Racer 250 EC, 2018.

In tubers collected from the plots where the active substance of fluorochloridon was applied, the presence of this active substance was not demonstrated (Figure 9).

**Figure 10:**
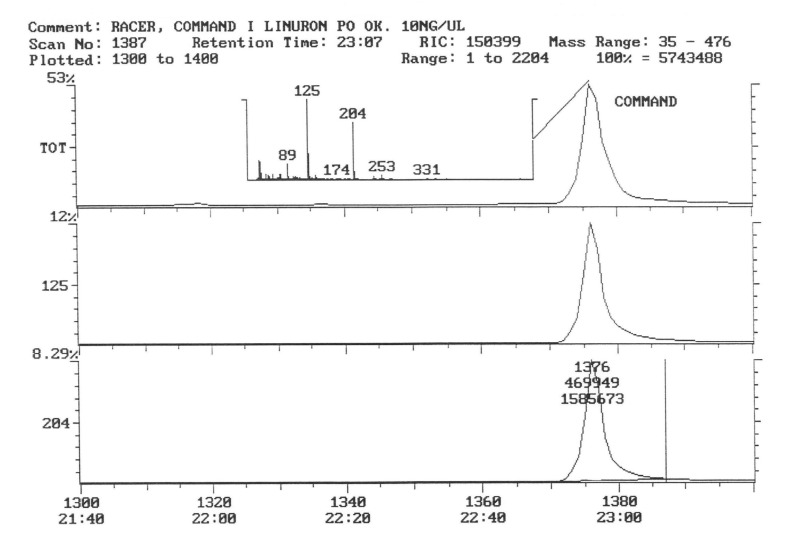
The chromatogram of the samples treated preparations with active substance clomazone in the first year of research.

In samples of tubers collected with the field sprayed with a mixture of herbicides (Afalon 450 SC + Command 480 EC) containing the active substances linuron and clomazone, one substance with the characteristic ion 204 was detected (Figure 10). This indicates the presence of clomazone. The concentration of clomazone was calculated from the chromatogram and was 0.0002 mg × kg^−1^ of a fresh sample of potato tubers. This content is significantly lower than the MRL [51].

**Figure 11:**
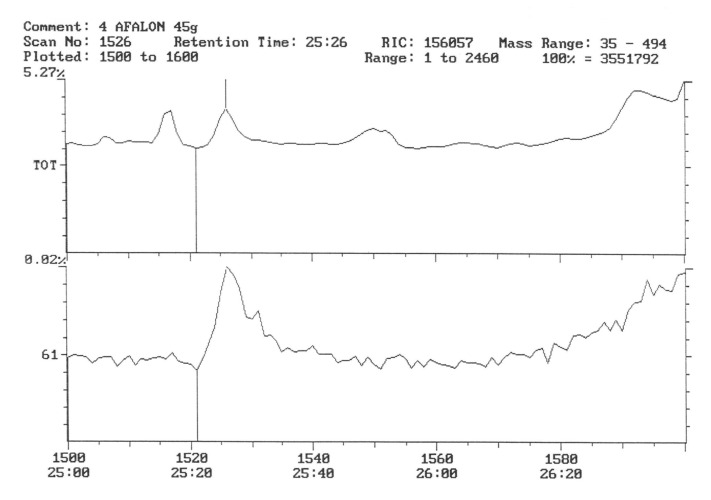
The chromatogram of the sample of potato-treated preparation with active substance linuron in the third year of research.

In the third year of the study of the pesticide, residues in potato tubers depended on the herbicide used in the cultivation. In the potato tubers of the combination sprayed with the preparation, the active substance of which was linuron, this substance was not detected (Figure 11).

**Figure 12:**
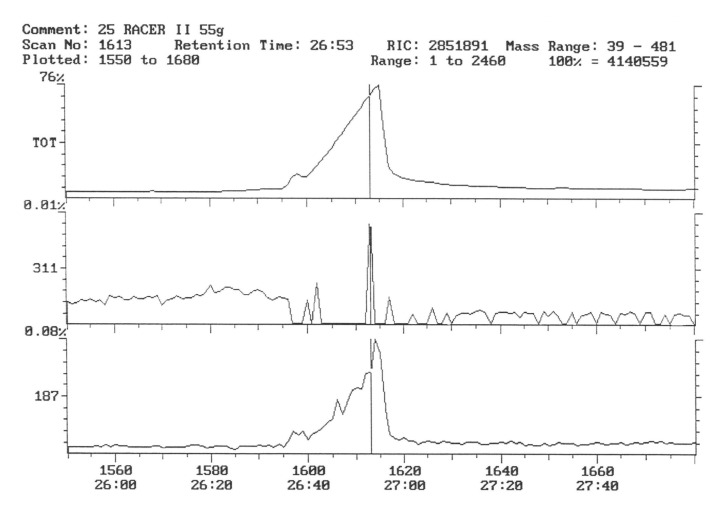
Chromatogram of the sample treated with the preparation, the active substance of which was fluorochloridon, in the second year of the study.

**Figure 13:**
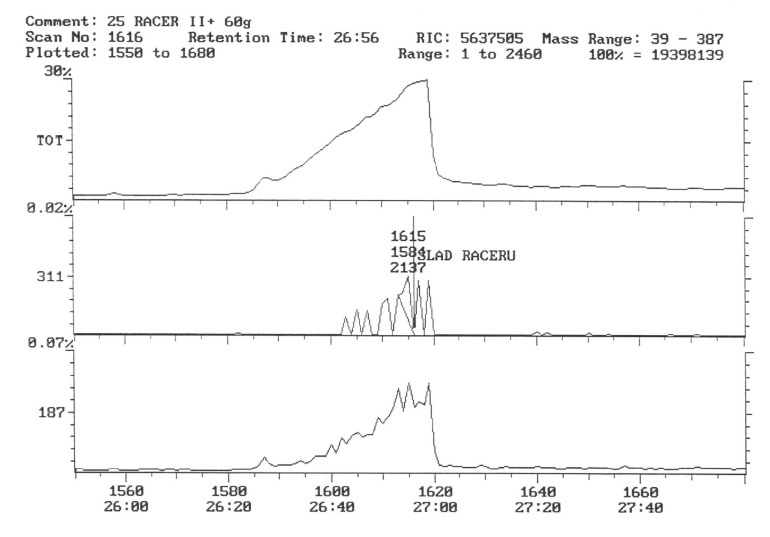
The chromatogram of the samples treated preparation with fluorochloridon in the third year of research.

The characteristic 311 ion was detected in the facilities where the preparation with fluorochloridon was used as an active substance, which indicates the presence of trace amounts of this compound (Figure 12 and Figure 13). The concentration of this preparation was calculated based on external calibration and was a 0.00024 mg.kg^−1^ fresh sample of potato. This content, however, is well below the MRL [53].

**Figure 14:**
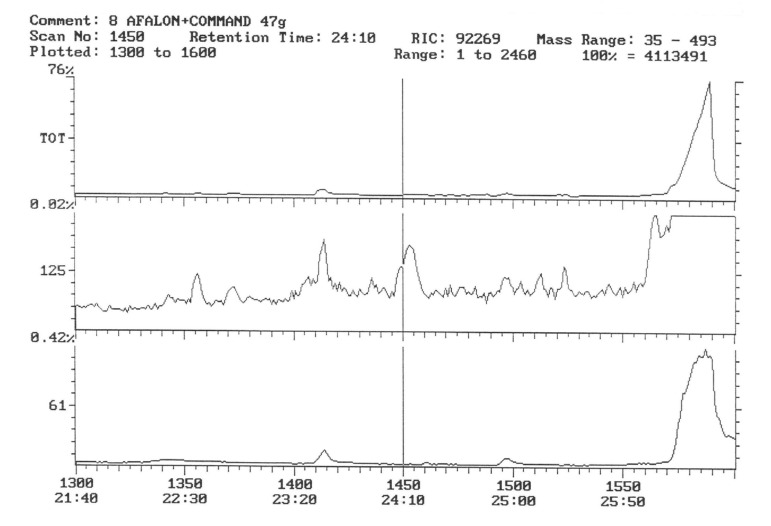
The chromatogram of the sample treated preparation with active substances linuron and clomazone in the second year of research.

**Figure 15:**
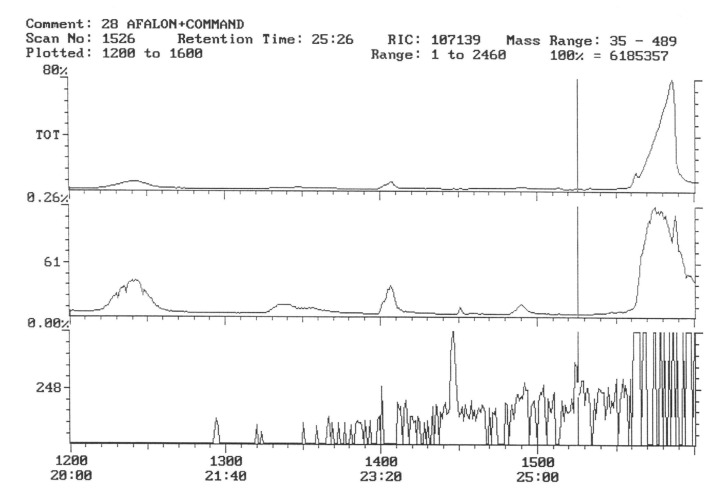
The chromatogram of the samples treated preparation with active substances linuron and clomazone in the third year of research.

Potato tubers from objects sprayed with a mixture of preparations containing linuron and clomazone were analyzed for the content of these active substances. However, they were not present in the test samples (Figure 14 and Figure 15). 

## 4. Discussion

### 4.1. Management of Potato Cultivation

Potato plants have a complex light perception system that allows them to adapt and optimize their yield and metabolism based on specific conditions. Light influences many developmental and physiological processes, including germination, flowering, growth direction, photosynthesis, and photomorphogenesis [16,23]. In the case of photosynthesis, pigments such as chlorophyll a and b and carotenoids are responsible for the use of visible light or photosynthetically active radiation, absorbing light in two spectral regions, blue (B: 400–500 nm) and red (R: 600–700 nm) [23]. In the case of photomorphogenesis, i.e., processes related to morphological and physiological changes, at least four main classes of photoreceptors are used: phytochromes operating mainly in the red/far-red wave range, blue and UVA-reactive cryptochromes, blue-reactive phytotropins (phototropism), and UVB photoreceptors [16]. A wide variety of spectral modification techniques are used under protected crop structures (i.e., greenhouses, plastic nets, or tunnels) that combine physical protection and differentiated solar radiation that can promote the desired light-regulated physiological responses to improve the commercial production of various plant species arable crops, including of potato. This species is one of the basic edible plants in Europe and in the world, and protected cultivation is practiced in order to overcome biotic and abiotic stresses [23]. In particular, potato production under ‘flat’ covers enables growers to alter light quality or photobiology by manipulating different wavelengths to modify the morphological characteristics of potato plants based on their specific light requirements [6].

Perforated PE-sheeting perfectly transmits the sun’s rays, but is practically impermeable to water vapor. The air closed between the soil and the foil heats up strongly on a sunny day. Air temperature differences between the open area and the area covered with foil can reach up to 15 °C, depending on the intensity of sunlight. According to Wadas et al. [55], at noon, on a sunny day, a small layer of air above the soil heats up to 35–40 °C. High temperature is usually accompanied by a relative air humidity of 95–100%. Although such a set of conditions is short-lived and generally does not harm the developing potato sprouts in the soil, it may expose them to stress [56]. Moreover, such conditions greatly favor the development of weeds under cover. Perforated polyethylene film allows, on the one hand, better use of sunlight by manipulating the spectrum of radiation reaching the crops and promoting physiological responses to plant development, including leaf area index, chlorophyll and carotenoid content, tissue structure, tuberization, physiological disturbances, nutritional quality, etc. [16,57,58]. On the other hand, it causes an excessive increase in temperature and solar radiation during potato production under such a cover to reduce the level of ß-carotene, causing changes in the photosynthetic apparatus up to sunburn and an increase in solanine accumulation in tubers, [59,60]. However, in the conducted studies, no significant effect of the application of PE-sheeting on damage to the potato, or monocotyledonous and dicotyledonous weeds, as well as their fresh and dry mass and number, was found. On the other hand, a significant impact of cultivation management on the floristic composition of weeds was found. In cultivation under PE-sheeting, a significant reduction in the number of burdensome species such as *Echinochloa cruss-galli*, *Setaria glauca,* and *Viola arvensis* was observed, and an increase in the number of species such as *Anthemis arvensis* and *Cirsium arvense*. Similar results were obtained by Wadas et al. [55].

### 4.2. Weed Infestation

Weeds are an important problem in potato cultivation under PE-sheeting, but research to improve weed management is limited. One of the objectives of the research was to conduct phytosociological studies of weed communities in the cultivation of a very early potato variety under a PE-sheeting. The conducted research showed that the soil coverage with weeds was generally low, which was confirmed by the studies by Pałys [56], which showed that the soil cover with weeds, in the soil and climatic conditions of the south-eastern part of Poland, is within 5–25%. As mentioned earlier, potato plants grown under cover, under shade conditions, or with a low R: FR ratio suffer from shade avoidance reactions that can affect several phytohormones that are responsible for the growth and reallocation of plant resources, leading to plant survival in crowded communities [57,58]. However, the cost of this shadow avoidance reaction is, inter alia, down-regulation of the jasmonic acid pathway, which in turn reduces the plant’s defense response to insect pest attack, thereby exposing crops to higher levels of weed infestation, as previously documented for various crops [55,59].

Twenty weed species are identified in the dicotyledonous class and seven in the monocotyledonous class; the most weeds were from the *Poaceae, Chenopodiaceae, Asteraceae*, and *Polygonaceae* families, and these showed the greatest importance in the community. Similar observations in the conditions of the Lublin Region were made by Pałys [56] and Bojarszczuk et al. [57]. They observed up to 48 species of weeds in potato cultivation, but only eight species dominated in the potato field. The most abundant weed species were *Anthemis arvensis*, *Chenopodium album,* and *Echinochloa crus-galli*. The greatest numbers of *Setaria viridis* and *Setaria glauca* were found before the potato harvest, which is related to the developmental biology of these species. A similar study was undertaken by Villa et al. [58], who identified 35 weed species but from a larger group of families (17) and proved significant differences in phytosociological parameters between both weed species.

Management of potato cultivation significantly influenced the number of several species of weeds. Potato cultivation under the PE-Sheeting contributed to the reduction of *Echinochloa cruss-galli, Setaria glauca,* and *Viola arvensis* but increased the number of *Cirsium arvense* and *Anthemis arvensis* and also influenced the biology of weeds and their survival, which is confirmed by the studies by Pszczółkowski and Sawicka [59] and Wadas et al. [55].

*Echinochloa cruss-galli* as the most aggressive weed species was effectively reduced after the application of Afalon Dispersion 450 SC. The application of the Afalon Dispersion 450 SC + Command 480 EC herbicide mixture was also effective in reducing the number of *Echinochloa crus-galli*, while the use of the Racer 25 EC herbicide did not change the abundance of this species; on the contrary, there was a tendency to increase the number of *E. cruss-galli* in compared to mechanical weed control treatments. Boydston [60], Gugała and Zarzecka [61], and Ilić et al. [62] observed that the responses of segetal species to herbicides are individual, but they also depend on the atmospheric conditions.

Dry matter of weeds in our research had a significant impact on the weeds of the potato canopy, both in the traditional technology and under perforated polyethylene foil covers. This is confirmed by the research of Wadas et al. [55]. Regression analyses carried out by Pszczółkowski and Sawicka [59] show that the air-dry mass of weeds is a more accurate measure determining the threshold values of weed infestation in crops. Gugała et al. [61] and Zarzecka et al. [63] were stating that the harmfulness of weeds depends to a lesser extent on their number per area unit than on the biomass produced by them.

A significantly greater number of weeds and their rich floristic composition were found in a warm and dry year than in other cooler and wetter years. These results are consistent with the observations of Gugała et al. [61], Ilić et al. [62], and Zarzecka et al. [63]. The low effectiveness of Racer 250 EC in reducing the dry matter of weeds in conditions of high water deficit in the soil in April could result from the rate of absorption and decomposition of the herbicide active substance in the tissues of individual weed species, as according to studies by and Ilić et al. [62], resistant plants of a given species absorb less fluorochloridon than susceptible forms. In addition, fluorochloridon may interact with other preparations used in the protection of potatoes, and these compounds may have a direct effect on plant growth, regardless of their protective effect.

The weed species composition was most strongly modified by the weed control systems used. Afalon Dispersion 450 SC, used in a chemical weed control system, was highly effective in reducing weed species such as *Echinochloa crus-galli, Centaurea cyanus, Setaria glauca, Setaria viridisi,* and *Stellaria media* and had no or very little effect on *Anthemis arvensis, Polygonum convolvulus, Vicia tetrasperma*, and *Veronica hederaefolia*. Similar effectiveness of this herbicide was achieved by Zarzecka et al. [63] and Kołodziejczyk et al. [64].

Herbicide Racer 250 EC, used in our research in a chemical weed control system, was very effective in controlling the weed species *Polygonum convolvulus, Polygonum laphathifolium*, and *Viola arvensis* and completely ineffective against such species as *Echinochloa crus-galli, Chenopodium album, Cirsium arvensis, Setaria glauca, Setaria viridis,* and *Spergula arvensis*. Our own research is consistent with the reports of Wadas [55], Pszczółkowski et al. [65], and Gugała et al. [66].

The conducted research confirms the high effectiveness of herbicide mixtures used in potato cultivation. The use of the Afalon Dispersion 450 SC + Command 480 EC herbicide mixture in the cultivation of a very early potato variety confirms the high effectiveness of combating such weeds as *Chenopodium album, Cirsium arvense, Galeopsis tetrahit, Myosotis arvensis, Raphanus raphanistrum, Spergula arvensis, Veronica hederifolia*, and *Vicia tetrasperma*. However, the mixture of these herbicides showed little effectiveness against *Galium aparine, Polygonum lapathifolium, Stellaria media*, and *Viola arvensis*. High effectiveness of the mixture of these herbicides was also confirmed in the studies of Gugała et al. [66], which report high effectiveness of mixtures of both Command 480 EC and Command 360 EC with other herbicides in reducing weed infestation in winter oilseed rape.

The obtained results are not always consistent with other authors regarding the effectiveness of herbicides, because, as reported by Gugała et al. [61] and Zarzecka et al. [63], the effectiveness of weed control is determined not only by the dose of the preparation but also the date of their application, as well as meteorological and soil conditions. Physiological and biochemical studies show that the differences between herbicide-resistant and -sensitive plants depend on the absorption rate and the rate of decomposition of herbicides in plant tissues [67]. In the opinion of Urbanowicz [68] and Ilić et al. [62], the phenomenon of resistance to herbicides will intensify. The possibility of survival of many weed species on plantations after treatment has so far been attributed to inaccurate treatment or low-quality herbicides. Wu et al. [24] and Urbanowicz [68] proved that the strength of the phytotoxicity effect is determined by the morphological diversity of the leaves of individual potato cultivars and the number of stomata on the upper surface of the leaf blade. The cultivar with the highest degree of sensitivity is characterized by clustered leaves and the highest number of stomata on the upper surface of the leaf blade. The insensitive variety is characterized by loose leaves with a large amount of free space between the leaflets and as few stomata as possible.

A separate issue is the phytotoxicity of herbicides toward potato plants. In the conducted research, the use of herbicides did not exert a significant influence on the growth and development of potato plants. The phytotoxicity of herbicides on potato plants, after herbicide application, ranged from 1 to 2 degrees on the EWRC scale. Rimsulfuron caused greater phytotoxicity on potato plants than linuron or clomazone. The damage caused by rimsulfuron was observed as severe leaf chlorosis, while damage caused by clomazone appeared as browning or necrosis of the leaves. However, phytotoxicity was significantly reduced as early as 3 weeks after PRE, and no signs of damage were seen 5 weeks after herbicide application due to plant regrowth or herbicidal damage. Similarly, Urbanowicz [69] found that the damage to the potato caused by linuron and rimsulfuron was minor and disappeared as early as 3–4 weeks after treatment.

### 4.3. Pesticide Residues

Pesticide residues are determined by multicomponent methods. Michel [70] reports that an analysis of residues of linuron is mainly carried out by high-performance liquid chromatography. Hu et al. [71] also indicate high-performance liquid chromatography with diode detection as a method for the determination of pesticide residues, whereas determination by UV detection method is mainly used for the analysis of linuron [72]. Other phenyloureas can be determined by gas chromatography. One also used mass spectrometry, coulometry, and amperometry [72]. Crestani et al. [1], Hu et al. [71], and Almeida et al. [73] report that clomazone residues can be analyzed by gas chromatography. In the case of the carried out research, one assumed a uniform determination of herbicides residues by gas chromatography.

The residues of the active substances used in the conducted studies depended on the nature of the substances and the conditions in the years of the study. The values determined in tubers collected during harvest ranged from 0.006 mg × kg^−1^ of clomazone to 0.024 mg × kg^−1^ for fluorochloridon. In the clinical examinations of Jaźwa [74], average residues of fluorochloridon in the soil the day after the treatment were taking shape from 0.066 to 0.134 mg × kg^−1^, and immediately prior to harvest ranged from 0.008 mg × kg^−1^ to 0.012 mg × kg^−1^. Kapka-Skrzypczak et al. [72] and Jaźwa [74] report that a slow decay of certain substances in soil, expressed as half-life, indicates potential phytotoxic effects, but only under the condition that the subsequent plant is planted a short time after the treatment. In the study of Słowik-Borowiec et al. [75], of 95 tested potato samples for pesticides, only 4% of samples of residues of plant protection products below the permissible content were detected.

As physiological and biochemical research indicates [1,14,18,19], the differences between plants that are resistant and sensitive to the active substance depend on the rate of its absorption and distribution in plant tissues. It is believed that in a plant heavily damaged by the active substances, there is an inhibition of photosynthesis and assimilation. According to Sawicka et al. [19], the maturity of the tubers affects the accumulation of nutrients in potato tubers. Immature tubers may, in fact, have higher pesticide residues, as characterized by a greater concentration of phenolic compounds in them.

In potato tubers cultivated using herbicides, there may be residues of active substances. Their level is governed by appropriate Acts. Maximum levels of residues of active substances of plant protection products are given to the Commission regulation (UE) 2019/50 [51] and Commission regulation (UE) 2019/58 [52] on residue levels of pesticides. 

The size of herbicide residues in plants depends on the weather conditions. Drought during the growing season results in faster maturation of plants. Plants have less time to metabolize active substances and therefore the residues are formed in the generative parts of the plant. Conversely, in years with increased precipitation, where the vegetation is usually elongated, the residues accumulate in the vegetative parts of plants [8,20].

An important factor determining the size of the herbicides residues by Sadowski [8] is also the dose. This is not a linear relationship. Beyond a certain dose, there is a rapid increase in the residue. It has to do with the reduced metabolic abilities of active substances by plants [8,10]. Jaźwa [74] stated that the next day after the application of preparation Racer EC 250 in an amount of 1 dm^−3^ × ha^−1^, 0.25 kg × ha^−1^, that is a dose half smaller than the recommended, fluorochloridon residues in the soil were 0.104 ± 0.069 mg × kg^−1^. These residues disappeared according to the exponential equation Pt = 0.1120^−0.0771t^ (R^2^ = 0.98) and on day 81 after application amounted to only 0.004 ± 0.003 mg × kg^−1^, representing approx. 12% of the starting residue. Similarly, based on residues found in soil samples and on the identification of the residue (actual dose: 0.25 kg × ha^−1^) on the recommended dosage of 0.5 kg × ha^−1^, one determined the expected course of linuron decay (Pt = 0.2243^−0.0771t^ ). The residues found in the soil samples were, respectively, P0 = 0.1122 mg × kg^−1^ to P0 = 0.130 mg × kg^−1^. Such high residues immediately after the treatment may indicate that for the weeding therapy of potato plantation, preparation Racer 250 SC was used at the recommended dose of 0.5 kg × ha^−1^, not as given, at a dose of 0.25 kg × ha^−1^, and the decay of linuron is conducted in accordance with the prescribed exponential equation Pt = 0.1122^−0.0771^. According to the author, the residues of fluorochloridon, 24 days after application, are at the level of 0.013 ± 0.007 mg × kg^−1^, and then are reduced, and at maturity, there is no evidence of residues of fluorochloridon above DGO, and any residues do not exceed 4% of the MRL.

Variable weather affects the size of the residues, especially in the first months after the application of the herbicide. In the first two weeks after the application of the herbicide, the content of the active substance increases, and it is associated with the collection of plants. At the same time in the plant, detoxification processes of the active substance of the herbicide start to occur. The dynamics of these processes determine the size of residues. After the period of their growth, due to the detoxification, there is a decrease in the levels of residues of active substances [8,70].

The detection of residues of herbicides does not mean that they are always present. They do not always reach maximum values. The size of the residues and the probability of their occurrence also depend on the individual varietal response, e.g., some potato varieties are particularly sensitive to the applied herbicides on their crops. It is associated with a stronger absorption of the active substance, and simultaneously, with impaired ability to metabolize it. This increases the level of residues [8,10,70].

The residues of active substances of tested herbicides in the basic raw products suggest that farmers use them in accordance with the principles of GAP (Good Agricultural Practice) and, as a result, with few exceptions, are contained below the MRL, while in most cases in Poland, they exceed the permissible level of 0.01 mg × kg^−1^ established for foods intended for infants and young children. Linuron, calculated for intake of 0.19 kg of potato, does not exceed 8.7% of the ADI for children and 1.8% of the ADI for adults, and for fluorochloridon, respectively, 1.6% and 0.17%, of the ADI, while one-time intake of linuron and fluorochloridon, according to Jaźwa [74], at the assumed consumption of carrots 1 kg, calculated for the highest detected residues of linuron, does not exceed 16.5% of the ARfD for children and 8.9% of the ARfD for adults, and for fluorochloridon it is, respectively, 3.3% and 16% of the ARfD. In the light of the recent knowledge in this field, the residues should not cause health problems either in children or adult consumers of potato [2].

In Poland, pesticide residues in plants consumed by humans and animals have been studied for many years by the laboratories of the Institute of Plant Protection. These studies include active substances and their derivatives (insecticides, fungicides, and herbicides) present in vegetables and fruit grown in the country [74,76,77]. This allows the determination of the risks to human health arising from the presence of pesticide residues [10,72,76].

High effectiveness of chemical methods of plant protection against herbicide-resistant weeds can be obtained by using a properly selected agent at the right time and weather conditions that favor their action, e.g., air temperature below 10 °C is not conducive to the effective operation of foliar herbicides. Low humidity, in turn, weakens the penetration of the herbicide through the leaf tissue of weeds, and low soil moisture inhibits the germination of weed seeds and absorption of the active ingredient of the herbicide. Late application of the herbicide, when the target weed species are already in advanced stages of development, gives a chance to survive the oldest species that are at this point more resistant [17,19]. The resistance of weeds to herbicide causes increasing losses, as well as environmental pollution by chemical means. One can prevent the formation of resistance of weeds to herbicides by corresponding crop rotation, the use of not only the mechanical methods of maintenance of crops but also chemical application of herbicides with different mechanisms of action and, if necessary, the use of mixtures thereof [19,22].

In the analyzed samples, from the first year of the study, residues of linuron or fluorochloridon were not detected. However, there was the presence of clomazone, the concentration of which amounted to 0.0002 mg × kg^−1^. This is well below the MRLs set by the Commission regulation (UE) 2019/50 [51], which is 0.02 mg × kg^−1^. In the samples from the third year, residues of linuron were not also detected. No clomazone was also observed in them. In contrast, trace amounts of the active fluorochloridon (0.00024 mg × kg^−1^) were found, where the MRL was 0.1 mg × kg^−1^. The determined values are much lower than the MRL (maximum level) [53]. In the case of linuron, its contents may be zero or so low that it becomes undetectable for this method.

The technology of growing very early potato varieties under “flat” cover with the use of soil herbicides just after planting the potato turned out to be safe. A very early harvest of tubers, after 55 days of planting, did not affect the content of residues of active substances of the used herbicides. Residues that could be determined by this method were detected in only a few test samples, but only in trace amounts. The detected amount of fluorochloridon was more than 400 times smaller than the MRL, as in the case of clomazone, whose indicated content was 250 times smaller than the MRL. Kucharski and Urbanowicz [78] did not find that any of the tested samples of potato tubers, regardless of earliness variety, exceeded the limit values of MRL for linuron.

The research of many authors [6,12,74,76,79] shows that there is no ideal herbicide for use in potato cultivation because almost all of them cause smaller or larger side effects. The selection of appropriate herbicides or mixtures thereof is dictated by their availability on the market price, the state weed of plantation, and the cultivation method [9,11,80].

The metabolism of xenobiotic and the regulation of related signaling pathways are, in the opinion of Wu et al. [24], the basis for the activation of xenobiotic toxicity. The chemopreventive effect of xenobiotic, including herbicides, may result from the regulation of the expression and activity of XMEs, as well as the reduction of the level of reactive metabolites and inhibition of a number of signaling pathways induced by xenobiotic [5,7,13,24,41,80]. Therefore, is the European regulation [81] compatible with the precautionary principle, since in its third and fourth chapters it exempts from analysis in the field of toxic pesticides (genotoxicity, carcinogenicity, and endocrine disorders) in the commercial mixtures that are placed on the market and to which consumers and the environment are exposed, obliged only to shortened tests always performed by applicants?

Both research hypotheses were verified, and it was proved that:

- the use of herbicides in cultivation under the PE-sheeting reduced weed infestation and at the same time did not damage plants of the very early potato variety,
- the pre-emergence application of herbicides contributed to the reduction of the residues of biologically active substances in potato tubers while maintaining their high efficiency.

The revised results will be a useful solution for growers for an effective selection of potato cultivars with increased resistance to herbicide residues [78,82]. These results can also be used to develop tools useful in the assessment of potato resistance to drought. Conducted research indicates the necessity of further monitoring of the content of residues of plant protection products in potato cultivation.

The progress that has been made in the technology of potato production for accelerated harvesting has meant that “young” potatoes hit the market in Central and Eastern Europe 2–3 weeks earlier and are already competing with potatoes imported from Egypt or Cyprus. The use of a new, innovative crop management system and weed management system will allow for the safe production of early potatoes in the country and their limited imports from countries with warmer climates, but not the raw material with poorer quality parameters (with a high content of pesticide residues and heavy metals in tubers), limiting the possibility of “dragging” of dangerous quarantine pests. However, it is necessary to constantly monitor the crops of early potato varieties in order to maintain food safety.

## 5. Conclusions

The technology of cultivation under covers “on the flat” with the use of herbicides before planting potatoes turned out to be safe in the cultivation of very early potato varieties harvested 55 days after planting.

The fresh and dry weight of weeds was most effectively reduced with the use of a chemical weed control system using the herbicide Afalon Dispersion 450 S.C. 

The application of chemical weed control with herbicides has shown a differentiated effect on the destruction of weeds:

- Afalon Dispersion 450 S.C. showed a high efficiency in reducing Echinochloa crus-galli, Setaria glauca, Setaria viridis, Centaurea cyanus, and Stellaria media, and showed no or weak activity against Anthemis arvensis, Polygonum convolvulus, Vicia tetrasperma, and Veronica hederifolia.
- Racer 250 EC was effective in reducing Polygonum convolvulus, Polygonum lapathifolium, and Viola arvensis and ineffective against Echinochloa crus-galli, Setaria glauca, Setaria viridis, Chenopodium album, Cirsium arvense, Spergula arvensis, and Myosotis arvensis.
- the use of a mixture of Afalon Dispersion 450 SC + Command 480 EC has shown high efficiency in reducing Chenopodium album, Raphanus raphanistrum, Vicia tetrasperma, Cirsium arvense, Galeopsis tetrahit, Spergula arvensis, Veronica hederaefolia, Myosotis arvensis: lapathifolium, Galium aparine, Viola arvensis, and Stellaria media.

The concentrations of the residues of active substances of herbicides detected in the soil were higher than those determined in potato tubers. Their level depended on the type of active substance, temperature, and humidity in the study years.

The residues of herbicides in tubers were absent or their amounts were so negligible that they did not pose a threat to human and animal health:

- no trace amounts of linuron were found,
- the detected residues of the herbicide Racer 250 EC (fluorochloridon) were at least 400 times lower than the EU standard,
- the amount of active clomazone detected was 250 times lower than the MRL.

The selection of a specific herbicide in the cultivation of early potato varieties grown under cover should be determined by the current condition and degree of weed infestation of the plantation, as this will ensure its high herbicidal effectiveness and will not lead to undesirable phenomena (the accumulation of weeds, development of resistance, and phytotoxic reactions).

Integration of the results with metabolic, genetic, and physiological data may be the basis for the ideal genotype of potato characterized by increased resistance to herbicides and at the same time a high yield.

Meteorological conditions modified the weight and number of weeds as well as their floristic composition. In dry summer conditions, the following were more numerous: *Anthemis arvensis, Stellaria media, Veronica hederifolia, Vicia tetrasperma,* and *Viola arvensis*. In a wet and cold year, the following were more common: *Cirsium arvense, Polygonum lapathifolium, Polygonum aviculare, Raphanus raphanistrum, Rumex acetosella, Spergula arvensis,* and *Vicia hirsuta*.

## Figures and Tables

**Figure 4:**
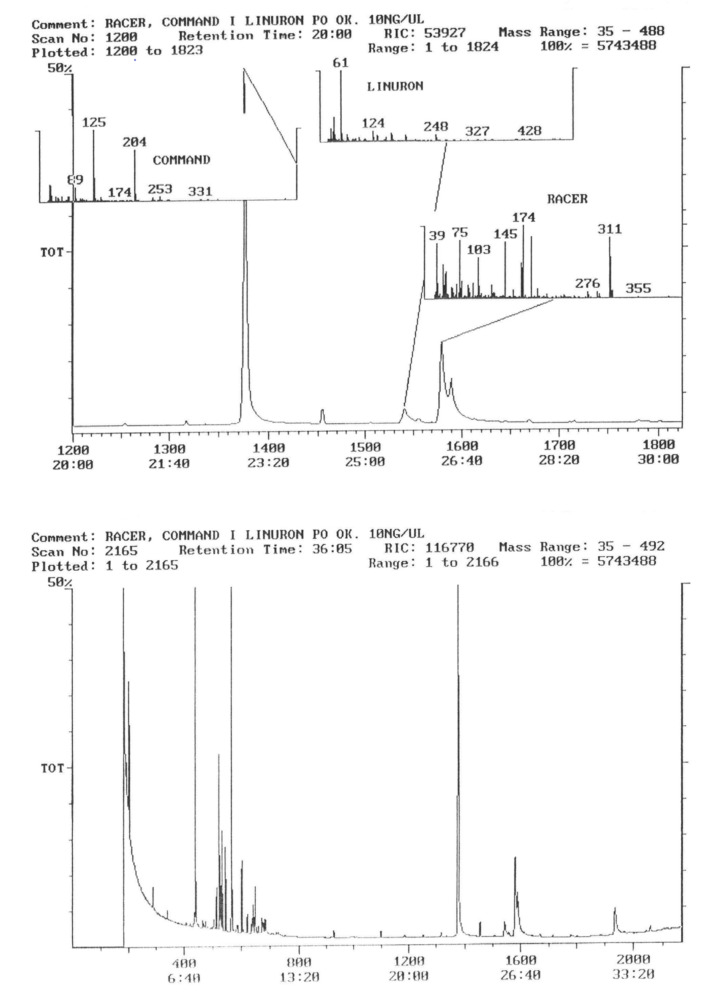
Chromatogram of the wavelengths of the individual active substances.

**Table 1:** Characteristics of selected active substances of herbicides.

Name of IUPAC	Characteristic
Linuron	
3-(3,4-Dichlorophenyl)-1-methoxy-1-methylurea N′-(3,4-Dichlorophenyl)-N-methoxy-N-methyl urea Methoxydiuron	Formula: C_9_H_10_Cl_2_N_2_O_2_ Molecular weight: t [MWpar, MWmet] (g/mol) 249.1 Melting point: 93–94 °C Water solubility, g/100 mL: <0.1 (does not dissolve) Vapor pressure, Pa at 24 °C: 0.002 Octanol/water partition coefficient as log Pow: 3.2
Fluorochloridon	
3-chloro-4-(chlorometylo)-1-[3-(trifluorometylo)fenylo]pirolidyn-2-on	Formula: C1_2_H_10_Cl_2_F_3_NO Molecular weight: t [MWpar, MWmet] (g/mol) 311.0091538 Melting point: 67, 65 °C Water solubility: 1.12 × 10^−4^ M (does not dissolve) Vapor pressure, Pa at 24 °C: 3,301.12 × 10^−6^ mmHg Octanol/water partition coefficient as log Pow: 3.2
Clomazone	
2-(2-chlorobenzyl)-4,4-dimethyl-oxazolidine-5-one	Formula: C1_2_H_14_ClNO_2_ Molecular weight: t [MWpar, MWmet] (g/mol) 239.7 Melting point: Solid at 20 °C with low melting temp. Water solubility, 1.212 g/L at 20 °C (pH 7) (Purity 99.0%) Water solubility is not pH-dependent Sum of evaporated radiolabeled clomazone was 6.9%

Source: [28,29,30].

**Table 2:** Characteristics of variety Lord.

Features	Characteristics
Maturity time	very early
Flesh color	yellow
Skin color	yellow
Mesh depth on a 9° scale	6.7
The size of the tubers on a 9° scale	8
Tuber shape	round oval
Cooking type *	AB
Resistance to late blight on a 9° scale	3
Resistance to PVYNTN on a 9° scale	7
Resistance to PLRV on a 9° scale	5.5
Resistance to common scab on a 9° scale	5
Storage resistance on a 9° scale	8
Resistance to storage diseases on a 9° scale	6

Source: [31]; * Culinary type A—salad; Culinary type B—general use; Culinary type C—slightly mealy. Intermediate culinary types—AB (salad/general use and CB (mealy/general).

**Table 3:** Doses of fungicides and insecticides in plant potato protection in 2016–2018.

2016	2017	2018
Fungicides		
Infinito 687.5 SC (propamocarb hydrochloride + fluopicolide) (625 g × dm^−3^ + 62.5 g × dm^−3^)–1.5 dm^3^ × ha^−1^ Ridomil Gold MZ 67.8 (mancozeb + metalaxyl) (640 g × dm^−3^ + 38.3 g × dm^−3^)–2 kg × ha^−1^	Acrobat MZ 69 WG (dimethomorph + mancozeb) (90 g × dm^−3^ + 600 g × dm^−3^)–2.0 kg × ha^−1^ Infinito 687.5 SC (propamocarb hydrochloride + fluopicolide) (625 g × dm^−3^ +62.5 g × dm^−3^)–1.5 dm^3^ × ha^−1^	Acrobat MZ 69 WG (dimethomorph + mancozeb) (90 g × dm^−3^ +600 g × dm^−3^)–2.0 kg × ha^−1^ Infinito 687.5 SC (propamocarb hydrochloride–625 g × dm^−3^, fluopicolide–62.5 g × m^−3^)–1.6 dm^3^ × ha^−1^
Insecticides		
Apacz 50 WG (clothianidin 500)–0.04 kg × ha^−1^ Proteus OD 110 (thiacloprid + deltamethrin) (100 g × dm^−3^+ 10 g × dm^−3^)–0.4 dm^3^ × ha^−1^	Actara 25 WG (thiamethoxam 250 g × dm^−3^)–0.08 kg × ha^−1^ Nuprid 200 SC (imidacloprid 200 g × dm^−3^) (0.15 dm^3^ × ha^−1^)	Actara 25 WG (thiamethoxam 250)–0.08 kg × ha^−1^ Proteus OD 110 (thiacloprid + deltamethrin) (100 g × dm^−3^ + 10 g × dm^−3^)–0.4 dm^3^ × ha^−1^

Source: own research.

**Table 4:** Physical and chemical properties of soil in Parczew (2016–2018).

Trait	Unit	Years			Average
		2016	2017	2018	
Granulometric composition					
Sand (mm)	%	66.85	67.23	66.81	66.96
Silt (mm)	%	30.52	30.10	31.13	30.58
Loam (mm)	%	2.63	2.67	2.06	2.45
Soil classification	Sandy loam (SL)				
Macronutrients					
P_2_O_5_	g 100 × g^−1^ of soil	20.1	18.9	24.0	21.0
K_2_O	g 100 × g^−1^ of soil	13.1	10.9	11.8	11.9
MgO	g 100 × g^−1^ of soil	7.8	7.0	6.3	7.0
Micronutrients					
Cu	mg × kg^−1^ of soil	7.51	4.92	8.99	7.02
Mn	mg × kg^−1^ of soil	318	337	166	274
Zn	mg × kg^−1^ of soil	40.1	56.7	41.1	46.0
Fe	mg × kg^−1^ of soil	3760	3925	3600	3762
B	mg × kg^−1^ of soil	7.24	5.28	6.04	6.17
Physico-chemical characteristics					
Humus content	%	0.94	1.06	1.03	1.02
pH	1M KCl	5.92	5.77	6.60	

Source: the results of own research were carried out at the Central Research Laboratory of the University of Life Sciences in Lublin and at the Chemical and Agricultural Station in Lublin.

**Table 5:** Distribution of precipitation and air temperatures as well as Sielianinov hydrothermal coefficient, during the growing season of potato plants, in 2016–2018, according to the data of the meteorological station in Uhnin (Poland).

Month	Precipitation (mm)			Average Air Temperature (°C)			The Value of the Sielianinov Hydrothermal Coefficient *		
	2016	2017	2018	2016	2017	2018	2016	2017	2018
April	43.3	127.5	163.3	7.1	4.6	9.9	0.7	3.3	2.0
May	160.8	244.8	125.0	15.8	13.9	13.9	1.6	2.8	1.4
June	51.2	67.9	114.5	16.6	17.0	17.6	0.7	0.9	1.4
July	109.4	305.9	102.8	17.1	18.0	18.0	1.4	3.7	1.2
August	83.5	69.0	160.7	17.5	17.7	15.8	1.1	0.9	2.2
Total	261.0	468.0	376.1	-	-	-	-	-	-

* Sielianinov hydrothermal coefficient was calculated according to the formula: $k=\frac{10\;P}{\sum \;t}$ [53] where: P—the sum of the monthly rainfall in mm, Σt—the monthly of air temperature > 0 °C.

**Table 6:** Degree of damage to mono- and dicotyledonous weeds and to crop plants on the EWRC (1–9°) scale ^a^.

Experimental Factors		Dicotyledonous Weeds							Monocotyledonous Weeds							Crop Plants						
		Terms of Observations *																				
		1	2	3	4	5	6	X	1	2	3	4	5	6	X	1	2	3	4	5	6	x
Cultivation Management **	A	5.1 ^a^	5.5 ^a^	8.2 ^a^	8.7 ^a^	8.8 ^a^	8.9 ^a^	7.5 ^a^	5.1 ^a^	5.5 ^a^	8.8 ^a^	8.7 ^a^	8.7 ^a^	9.0 ^a^	7.6 ^a^	1.0 ^a^	1.0 ^a^	1.0 ^a^	1.0	1.0 ^a^	1.0 ^a^	1.0 ^a^
	B	4.^9 a^	5.1 ^a^	8.3 ^a^	8.6 ^a^	8.9 ^a^	9.0 ^a^	7.5 ^a^	4.9 ^a^	5.2 ^a^	5.3 ^a^	9.0 ^a^	9.0 ^a^	9.0 ^a^	7.0 ^b^	1.8 ^a^	2.4 ^a^	1.8 ^a^	1.4 ^a^	1.2 ^a^	1.0 ^a^	1.6 ^a^
	HSD_0.05_	ns ****						ns	ns						0.4	ns						ns
Weed control systems ***	M	9.0 ^a^	8.8 ^a^	9.0 ^a^	9.0 ^a^	9.0 ^a^	9.0 ^a^	9.0 ^a^	9.0 ^a^	9.0 ^a^	8.0 ^a^	8.5 ^a^	9.0 ^a^	9.0 ^a^	8.8 ^a^	1.0 ^a^	1.0b	1.0 ^a^	1.0 ^a^	1.0 ^a^	1.0 ^a^	1.0 ^b^
	Af	4.8 ^a^	4.6 ^a^	9.0 ^a^	9.0 ^a^	9.0 ^a^	9.0 ^a^	7.6 ^b^	4.8 ^a^	4.6 ^a^	5.0 ^a^	8.7 ^a^	9.0 ^a^	9.0 ^a^	6.9 ^b^	1.6 ^a^	1.7a	1.4 ^a^	1.2 ^a^	1.1 ^a^	1.0 ^a^	1.2 ^a^
	R	2.9 ^a^	3.9 ^a^	8.0 ^a^	8.5 ^a^	8.9 ^a^	9.0 ^a^	6.9 ^b c^	2.9 ^a^	3.9 ^a^	8.0 ^a^	8.5 ^a^	8.9 ^a^	9.0 ^a^	6.9 ^b^	1.8 ^a^	2.3 ^a^	1.8 ^a^	1.4 ^a^	1.2 ^a^	1.0 ^a^	1.5 ^a^
	Af + C	3.3 ^a^	4.0 ^a^	7.3 ^a^	8.1 ^a^	8.6 ^a^	9.0 ^a^	6.7 ^b c^	3.3 ^a^	4.0 ^a^	7.3 ^a^	8.4 ^a^	8.6 ^a^	9.0 ^a^	6.8 ^b^	1.4 ^a^	1.6 ^a^	1.4 ^a^	1.2 ^a^	1.1 ^a^	1.0 ^a^	1.3 ^a^
	HSD_0.05_	ns						1.0	ns						0.8	ns						0.3
Years	2016	3.0 ^a^	3.7 ^a^	8.7 ^a^	9.0 ^a^	9.0 ^a^	9.0 ^a^	7.1b	3.2 ^a^	4.0 ^a^	7.0 ^a^	9.0 ^a^	9.0 ^a^	9.0 ^a^	6.9 ^b^	1.6 ^a^	2.2 ^a^	1.6 ^a^	1.2 ^a^	1.1 ^a^	1.0 ^a^	1.4 ^a^
	2017	4.7 ^a^	4.5 ^a^	8.5 ^a^	8.6 ^a^	8.6 ^a^	9.0 ^a^	7.3 ^b^	4.6 ^a^	4.8 ^a^	7.2 ^a^	8.6 ^a^	8.7 ^a^	9.0 ^a^	7.2 ^b^	1.4 ^a^	1.7 ^a^	1.5 ^a^	1.2 ^a^	1.1 ^a^	1.0 ^a^	1.3 ^a^
	2018	7.2 ^a^	7.7 ^a^	7.8 ^a^	8.6 ^a^	9.0 ^a^	9.0 ^a^	8.2 ^a^	7.3 ^a^	7.4 ^a^	7.1 ^a^	8.0 ^a^	9.0 ^a^	9.0 ^a^	8.0 ^a^	1.3 ^a^	1.2 ^a^	1.1 ^a^	1.1 ^a^	1.0 ^a^	1.0 ^a^	1.1 ^b^
	HSD_0.05_	ns						0.8	ns						0.6	ns						0.2
Average		5.0 ^c^	5.3 ^c^	8.3 ^b^	8.6 ^a^	8.9 ^a^	9.0 ^a^	7.5	5.0 ^c^	5.4 ^c^	7.1 ^b^	8.5 ^a^	8.9 ^a^	9.0 ^a^	7.3	1.4 ^b^	1.7 ^a^	1.4 ^b^	1.2 ^b^	1.1 ^c^	1.0 ^c^	1.3
HSD_0.05_		1.5							1.2							0.3						

* Observation dates from emergence of plants every 7 days; ** A—traditional technology, B—PE-sheeting; M—mechanical care; *** Af—Afalon 450 SC; R–Racer 250 EC, Af + C—Afalon 450 SC + Command 480 EC; **** not significant at *p* ≤ 0.05. The presence of the same letter index by the means (at least one) means that there is no statistically significant difference between them. The subsequent letter indices a, b, c, define the groups in descending order; ^a^ scale EWRC for weeds: 1—complete destruction of weeds; 9—no action on weeds’; scale EWRC for the cultivated plant: 1—no damage symptoms, 9—completely destroyed plants.

**Table 7:** Fresh and air dry mass of weeds (g × m^−2^).

Experimental Factors *		A Fresh Mass of Weeds				Dry Mass of Weeds			
		Observation Dates **							
		1	2	3	Average	1	2	3	Average
Cultivation management	Traditional	37 ^a^ ***	63 ^a^	203 ^a^	101 ^a^	7 ^a^	9 ^a^	92 ^a^	36 ^a^
	PE-sheeting	24 ^a^	66 ^a^	211 ^a^	100 ^a^	5 ^a^	11 ^a^	89 ^a^	35 ^a^
	HSD_0.05_	ns ****			ns	ns			ns
Weed control Systems	Mechanical	21 ^a^	55 ^a^	201 ^a^	92 ^b^	4 ^a^	9 ^a^	89 ^b^	34 ^b^
	Afalon	27 ^a^	53 ^a^	170 ^a^	83 ^b^	5 ^a^	9 ^a^	82 ^b^	32 ^b^
	Racer	36 ^a^	43 ^b^	270 ^a^	116 ^a^	7 ^a^	9 ^a^	112 ^a^	43 ^a^
	Afalon + Command	21 ^a^	72 ^a^	185 ^a^	93 ^b^	4 ^a^	15 ^a^	83 ^b^	34 ^b^
	HSD_0.05_	34			15	19			9
Years	2016	13 ^b^	58 ^b^	482 ^a^	184 ^a^	2 ^a^	11 ^a^	101 ^a^	38 ^b^
	2017	5 ^b^	15 ^c^	105 ^b^	42 ^c^	1 ^a^	2 ^a^	31 ^a^	11 ^c^
	2018	61 ^a^	95 ^a^	36 ^c^	64 ^b^	12 ^a^	18 ^a^	143 ^a^	58 ^a^
	HSD_0.05_	28			12	ns			7
Average		27 ^c^	56 ^b^	207 ^a^	96	6 ^b^	10 ^b^	92 ^a^	36
HSD_0.05_		12				7			

* Observation dates from emergence of plants every 7 days; ** 1—before the rows are closed, 2—after the rows are closed, and 3—before the potato is ripening; *** letter indicators at the means (significant groups) define the so-called homogeneous (statistically homogeneous) groups. The presence of the same letter index by the means (at least one) means that there is no statistically significant difference between them. The subsequent letter indices ^a, b, c,^ define the groups in descending order; **** ns—not significant at *p* ≤ 0.05.

**Table 8:** The number of monocotyledonous and dicotyledonous weeds in pcs. m^−2^.

Experimental Factors *		Monocotyledonous				Dicotyledonous				Total			
		Observation Dates **											
		1	2	3	X	1	2	3	X	1	2	3	X
Cultivation management	Traditional	47 ^a^ ***	20 ^a^	20 ^a^	29 ^a^	27 ^a^	22 ^a^	20 ^a^	23 ^a^	74 ^a^	42 ^a^	40 ^a^	52 ^a^
	PE-sheeting	35 ^a^	15 ^a^	16 ^a^	22 ^b^	22 ^a^	10 ^a^	20 ^a^	17 ^a^	57 ^a^	25 ^a^	36 ^a^	39 ^a^
	HSD_0.05_	ns **			3	ns			ns	ns			ns
Weed control systems	Mechanical	45 ^a^	20 ^a^	19 ^a^	28 ^b^	26 ^a^	12 ^a^	18 ^a^	18 ^b^	71 ^a^	32 ^b^	36 ^b^	46 ^b^
	Afalon	36 ^a^	11 ^b^	10 ^b^	19 ^c^	32 ^a^	22 ^a^	19 ^a^	24 ^a^	68 ^a^	33 ^b^	29 ^b^	43 ^b^
	Racer	43 ^a^	31 ^a^	30 ^a^	35 ^a^	25 ^a^	20 ^a^	26 ^a^	24 ^a^	68 ^a^	51 ^a^	56 ^a^	58 ^a^
	Afalon + Command	41 ^a^	12 ^b^	13 ^b^	22 ^c^	18 ^a^	12 ^a^	14 ^a^	15 ^b^	59 ^a^	23 ^b^	27 ^b^	36 ^c^
	HSD_0.05_	13			6	ns			5	19			9
Years	2016	23 ^b^	33 ^a^	43 ^a^	33 ^a^	9 ^c^	26 ^a^	38 ^a^	25 ^a^	33 ^b^	59 ^a^	81 ^a^	58 ^a^
	2017	19 ^b^	18 ^b^	6 ^b^	14 ^b^	22 ^b^	14 ^b^	10 ^b^	16 ^c^	41 ^b^	32 ^b^	16 ^b^	30 ^c^
	2018	81 ^a^	4 ^c^	4 ^b^	30 ^a^	44 ^a^	8 ^b^	9 ^b^	20 ^b^	125 ^a^	12 ^c^	13 ^b^	50 ^b^
	HSD_0.05_	10			4	10			4	16			7
Average		41 ^a^	18 ^b^	18 ^b^	26	25 ^a^	16 ^b^	19 ^b^	20	66 ^a^	35 ^b^	37 ^b^	46
HSD_0.05_		4				4				7			

* Observation dates from emergence of plants every 7 days; ** 1—before the rows are closed, 2—after the rows are closed; 3—before the potato is ripening; *** letter indicators at the means define the so-called statistically homogeneous groups. The presence of the same letter index by the means (at least one) means that there is no statistically significant difference between them. The subsequent letter indices a, b, c, define the groups in descending order.

**Table 9:** Species composition and number of monocotyledonous and dicotyledonous weeds in pcs. m^−2^.

Experimental Factors		*Agropyron repens*	*Apera spica-venti*	*Avena fatua*	*Echinochloa crus-galli*	*Poa a* *nnua*	*Setaria glauca*	*Setaria viridis*	*Anagalis arvensis*	*Anthemis arvensis*	*Centaurea cyanus*	*Chenopodium album*	*Cirsium arvense*	*Galium aparine*	*Galeopsis tetrahit*	*Myosotis arvensis*	*Polygonum convolvulus*	*Polygonum lapathifolium*	*Poligonum aviculare*	*Stellaria media*	*Spergula arvensis*	*Veronica hederifolia*	*Vicia hirsuta*	*Vicia cracca*	*Vicia tetrasperma*	*Viola arvensis*	*Raphanus raphanistrum*	*Rumex acetosella*
Cultivation management **	T	0.6	0.2	0.1	25.4	0.1	1.6	1.2	0.1	5.3	0.8	5.5	0.3	0.0	0.0	0.3	1.7	0.2	0.1	0.5	0.5	0.8	0.1	0.1	0.2	1.1	3.0	0.3
	PE	0.8	0.2	0.1	19.4	0.1	0.8	1.1	0.1	5.9	0.8	4.5	1.1	0.0	0.0	0.3	1.8	0.2	0.3	0.6	0.5	0.6	0.1	0.0	0.3	0.7	3.3	0.1
	_HSD0.05_	n *	n	n	5.6	n	0.4	n	n	0.4	n	n	0.6	n	n	n	n	n	n	n	n	n	n	n	n	0.4	n	n
Weed control system ***	A	0.7	0.1	0.1	24.4	0.2	1.5	1.4	0.1	3.0	1.0	5.3	0.6	0.0	0.2	0.2	1.9	0.1	0.1	0.6	0.4	0.4	0.1	0.0	0.3	0.6	4.4	0.1
	B	0.5	0.1	0.0	16.5	0.0	0.6	0.7	0.1	8.8	0.6	4.0	0.6	0.0	0.1	0.4	2.4	0.3	0.2	0.4	0.6	1.4	0.1	0.1	0.6	1.1	2.9	0.3
	C	0.5	0.3	0.1	29.8	0.1	1.9	1.6	0.2	6.7	0.8	9.0	1.2	0.0	0.0	0.4	1.0	0.0	0.1	0.6	0.9	0.6	0.0	0.0	0.3	0.6	2.9	0.2
	D	0.5	0.2	0.1	18.9	0.0	0.8	1.0	0.1	3.9	0.9	1.9	0.5	0.1	0.0	0.2	1.9	0.4	0.2	0.9	0.1	0.3	0.0	0.1	0.1	1.3	2.6	0.1
	HSD_0.05_	0.5	n	n	5.6	n	0.4	0.7	0.1	2.3	n	3.1	0.6	0.1	0.1	0.2	1.1	0.2	n	0.4	0.7	0.5	n	n	0.3	0.5	n	n
**Years**	2016	0.3	0.5	0.3	25.8	0.3	3.1	2.9	0.2	13.5	1.2	3.4	0.1	0.0	0.0	0.6	1.1	0.0	0.0	1.2	0.3	2.0	0.0	0.0	1.0	1.3	0.0	0.0
	2017	1.6	0.0	0.0	12.5	0.0	0.6	0.0	0.0	0.0	0.0	2.7	2.1	0.1	0.0	0.1	0.2	0.5	0.5	0.0	1.1	0.0	0.0	0.1	0.0	0.2	8.7	0.5
	2018	0.3	0.0	0.0	29.0	0.0	0.0	0.5	0.1	0.0	1.0	9.1	0.0	0.0	0.2	0.2	4.1	0.1	0.0	0.6	0.1	0.0	0.2	0.0	0.0	1.2	0.9	0.0
	HSD_0.05_	0.4	0.2	0.2	4.4	0.2	0.3	0.5	0.1	1.8	0.4	2.5	0.4	0.1	0.1	0.2	0.9	0.2	0.1	0.3	0.5	0.4	0.1	0.1	0.2	0.4	1.7	0.3
Observation dates ****	I	0.9	0.1	0.0	39.9	0.0	0.0	0.0	0.1	4.1	0.7	7.8	0.1	0.0	0.1	0.1	3.2	0.2	0.1	0.4	0.1	0.3	0.2	0.0	0.4	1.1	6.1	0.2
	II	0.6	0.1	0.1	17.1	0.3	0.0	0.8	0.0	6.3	0.8	3.8	1.3	0.0	0.0	0.0	0.5	0.1	0.3	0.2	0.1	0.8	0.0	0.1	0.2	0.4	2.9	0.0
	III	0.7	0.3	0.2	10.2	0.1	3.7	2.7	0.2	6.4	0.9	3.5	0.8	0.1	0.0	0.7	1.7	0.2	0.2	1.3	1.4	0.9	0.0	0.0	0.4	1.2	0.6	0.3
	HSD_0.05_	n	0.2	0.2	4.4	0.2	0.3	0.5	0.1	1.8	n	2.5	0.4	0.1	n	0.2	0.9	n	0.1	0.3	0.5	0.4	0.1	0.1	n	0.4	1.7	n
**Average**		0.7	0.2	0.1	22.4	0.1	1.2	1.2	0.1	5.6	0.8	5.0	0.7	0.0	0.0	0.3	1.8	0.2	0.2	0.6	0.5	0.7	0.1	0.0	0.3	0.9	3.2	0.2

n *—not significant at *p* ≤ 0.05; ** T—traditional technology; PE—PE-sheeting. *** A—mechanical; B—Afalon Dispersion 450 SC; C—Racer 250 EC; D—Afalon Dispersion 450 SC + Command 480 EC; **** I—before the rows are closed, II—after the rows are closed, III—before harvest.

**Table 10:** Average residues of herbicides in soil materials (mg × kg^−1^).

Active Substance	Years			Average
	2016	2017	2018	
Linuron	0.0160 ± 0.0031 ^a,^*	0.0140 ± 0.0032 ^a^	0.0101 ± 0.0025 ^a^	0.013 ^a^
Fluorochloridon	0.0150 ± 0.0021 ^b^	0.0132 ± 0.0020 ^b^	0.0080 ± 0.0007 ^b^	0.012 ^a^
Clomazone	0.0081 ± 0.0003 ^c^	0.0007 ± 0.0001 ^b^	0.0050 ± 0.0002 ^b^	0.004 ^b^
Average	0.0130 ^a^	0.009 ^b^	0.0077 ^b^	
HSD_0.05_	Herbicides (H)—0.006; years (Y)—0.006, H × Y—0.018			

± standard deviations; * letter indicators at the means define the so-called statistically homogeneous groups. The presence of the same letter index by the means (at least one) means that there is no statistically significant difference between them. The subsequent letter indices ^a, b, c^, define the groups in descending order.

## Abbreviations

ADI	Acceptable Daily Intake
ArfD	Acute Reference Dose
BBCH scale	Biologische Bundesanstalt, Bundessortenamt, Chemische Industrie
DHP	dihydropteroate synthase inhibitor
EWRC	European Weed Research Council
EPSP	EPSP synthase inhibitors–5-enolpyruvate shikimic 3-phosphate synthase
HLD	High-density lipoprotein
LDL	Low-density lipoprotein
LHC	light energy collecting complex
MRL	Maximum Residue Level
PE-sheeting	Polyethylene sheeting
